# Patient characteristics, treatment patterns, and survival across estrogen receptor expression subgroups in HER2-negative breast cancer: a population-based cohort study

**DOI:** 10.1016/j.lanepe.2026.101826

**Published:** 2026-08-26

**Authors:** Qiao Yang, Ceren Boyaci, Ioannis Zerdes, Irma Fredriksson, Xinsong Chen, Johan Hartman, Balazs Acs

**Affiliations:** a Department of Oncology and Pathology, Karolinska Institutet, Stockholm, Sweden; b Department of Clinical Pathology and Cancer Diagnostics, Karolinska University Hospital, Stockholm, Sweden; c Theme Cancer, Karolinska University Hospital and Comprehensive Cancer Center, Stockholm, Sweden; d Department of Molecular Medicine and Surgery, Karolinska Institutet, Stockholm, Sweden; e Department of Breast-, Endocrine Tumors and Sarcoma, Karolinska University Hospital, Stockholm, Sweden

**Keywords:** Breast cancer, Estrogen receptor, ERmild expression, ER-intermediate

## Abstract

**Background:**

Estrogen receptor (ER) low (1–9%) breast cancer (BC) often behaves like triple-negative BC, while ERhigh (≥60%) disease has a more favorable prognosis. However, tumors with ER expressing 10–59% represent a poorly defined patient population. In this nationwide cohort study, we aimed to describe real-world patient characteristics, treatment patterns and survival across the entire ER spectrum in Human Epidermal growth factor Receptor 2 (HER2)-negative BC.

**Methods:**

We conducted a population-based cohort study of all women diagnosed with HER2-negative BC in Sweden (2007–2023), based on prospectively collected data. Tumors were stratified into five ER subgroups: ER^0%^ (ERzero), ER^1–9%^ (ERlow), ER^10–29%^ (ERmild), ER^30–59%^ (ERmod), and ER^60–100%^ (ERhigh). We evaluated overall survival (OS) by ER subgroups and treatment (chemotherapy [CT] and endocrine therapy [ET]) using Kaplan–Meier analysis and multivariable Cox regression.

**Findings:**

We included 75,211 patients. Compared to the ERhigh subgroup, both the ERmild (adjusted HR [aHR] = 1.59, 95% confidence interval [CI]: 1.31–1.93) and ERmod (aHR = 1.31, 95% CI: 1.17–1.47) subgroups had significantly worse survival (both adjusted P [aP] < 0.001). Notably, ERmild patients had survival outcomes and molecular characteristics similar to those of ERlow and ERzero patients. Patients with ERmild (aHR = 0.46, 95% CI: 0.23–0.94, aP = 0.033) and ERmod (aHR = 0.60, 95% CI: 0.42–0.85, aP = 0.0046) tumors, CT + ET was associated with a lower risk of death than ETonly.

**Interpretation:**

Our findings highlight the importance of assessment of ER-low, mild and moderate tumors (1–59%). Reporting ER as a percentage can provide additional biological insight in clinical decision-making.

**Funding:**

This study received no external funding.

**Research in context**

**Evidence before this study**

We searched PubMed for articles published until December 2025, using terms “estrogen receptor low”, “ER 1–9%”, “ER 10–29%”, “intermediate ER expression”, “breast cancer survival”, and “treatment patterns”. Existing evidence confirmed that ERlow (1–9%) breast cancer (BC) behaves similarly to triple-negative disease, with uncertain benefit from endocrine therapy. While the ≥10% threshold is used to define ER-positive disease, robust population-based data on the prognosis and optimal treatment for tumors with intermediate ER expression (10–59%) were lacking, particularly for the 10–29% subgroup. No previous study had comprehensively evaluated the survival continuum and treatment efficacy across ER subgroups in a large real-world cohort.

**Added value of this study**

This prospectively collected, population-based retrospective cohort study of 75,211 patients with HER2-negative BC provides a detailed characterization of the ER continuum. We demonstrate that both ERmild (10–29%) and ERmod (30–59%) tumors have significantly worse survival than ERhigh (60–100%) tumors, and that ERmild tumors have a prognosis and molecular characteristics similar to ERlow and ERzero tumors. Most importantly, for patients with ERmild and ERmod tumors, adding chemotherapy to endocrine therapy was associated with a lower risk of death compared to endocrine therapy alone.

**Implications of all the available evidence**

The cumulative evidence indicates that the estrogen receptor expression is a continuous, rather than binary, prognostic and predictive biomarker. For clinical practice, these findings suggest that the current ≥ 10% cutoff is insufficient for therapeutic decisions in all BC patients. This study supports recognizing specific ER subgroups and calls for the routine reporting of ER percentages to guide tailored treatment strategies, especially for patients with mild and moderate ER expressing breast cancer.

## Introduction

Estrogen receptor (ER) positive breast cancer (BC) represents the largest subgroup of breast tumors. ER status is a powerful prognostic factor and strongly predictive for the benefits of endocrine treatment. The ASCO/CAP guidelines define ER-positivity as immunohistochemical nuclear staining of ER in ≥1% of tumor cells; consequently, the cutoff does not adequately capture the biological heterogeneity among those considered positive.^1^ The low cut-off value ensures patients with minimal ER expression are not excluded from potential endocrine therapy benefit, even if in the presence of problems in preanalytical/analytical phases. However, this approach has been questioned because the scientific evidence is insufficient to support the benefits of endocrine therapy in populations with lower ER expression.^2^^,^^3^ In Sweden, the old definition of ER-positivity, ≥10%, has been kept.^4^

Recently, clinical attention has focused on the ER-low subgroup (1–10%), which was recognized by ASCO/CAP in 2020.^5^ Since then, several studies have analyzed the benefits of endocrine therapy in ER-low cases. While some studies suggested a potential benefit, others could not confirm it, leaving the clinical relevance uncertain in practice.^6^ Molecular characterization has further provided insights, such as Higgins et al. reported that 82.6% of ER-low/Human Epidermal growth factor Receptor 2 (HER2) negative tumors in the Cancer Genome Atlas cohort were basal-like, whereas only 8.6% were luminal, proportions clearly differing from tumors with ER expression >10%.^7^

However, the clinicopathological and prognostic characteristics of potential intermediate ER subgroups remain poorly defined, as most have focused on ER-low^2^ or ER-high categories, while intermediate groups have received less attention. To address this knowledge gap, we aimed to describe real-world patient and tumor characteristics, treatment patterns and overall survival across ER subgroups in a comprehensive Swedish population-based cohort of patients with HER2-negative BC.

## Methods

### Study design and data source

This nationwide, population-based, prospectively collected retrospective cohort study aimed to investigate tumor characteristics, treatment patterns, and overall survival (OS) trends across different estrogen receptor (ER) subgroups in women with Human Epidermal growth factor Receptor 2 (HER2)-negative breast cancer (BC) in Sweden. The study utilized data from the Swedish National Quality Register for Breast Cancer (NKBC). The NKBC, linked to the mandatory National Swedish Cancer Register, has completeness exceeding 99% for BC cases (using the mandatory National Swedish Cancer Register as the gold standard), and its variables demonstrate high agreement with source data (with <5% missing values).^8^

### Study population

**Fig. 1:**
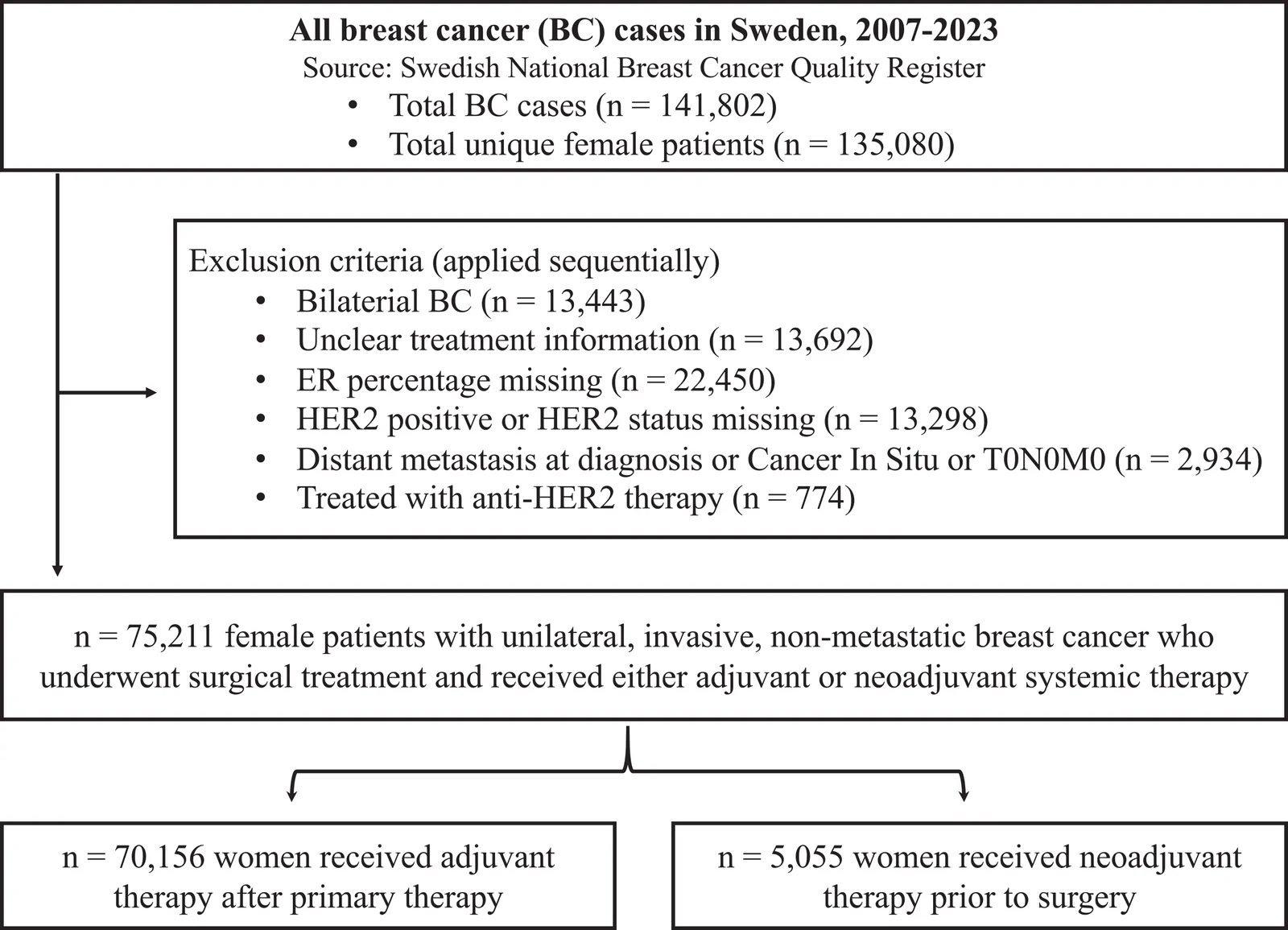
**Patient selection flowchart**. Diagram illustrating the sequential application of inclusion and exclusion criteria to derive the final study population from the Swedish National Quality Register for Breast Cancer (NKBC). Abbreviation: BC, breast cancer. Note: Nineteen patients initially registered as T0N0M0 were excluded from the cohort; however, they were reclassified as T1N0M0 in accordance with updated staging guidelines.

This study included all women diagnosed with primary, invasive, non-metastatic, HER2-negative BC in Sweden between January 2007 and December 2023. From the initial 141,802 BC cases, we first excluded those with bilateral disease (n = 13,443 cases). Subsequent exclusions were applied to ensure a clear and analyzable cohort (shown in Fig. 1): patients with unclear treatment information (n = 7112), missing date of surgery (n = 1725), missing pre-treatment information required for the identification of the neoadjuvant setting (n = 4730), and those with implausible time gaps between diagnosis and surgery (i.e. n = 45 surgery >1 month before diagnosis for the adjuvant setting or n = 80 > 12 months after diagnosis). In total, additional 13,692 BC cases were excluded, with the primary reason summarized as “Unclear treatment information” for flow chart purposes. The final cohort consisted of 75,211 patients with primary, invasive, and non-metastatic HER2-negative BC, all of whom had complete data on ER percentage, treatment, and follow-up.

### Variables

Key biomarkers (ER, progesterone receptor [PR], HER2 status, and Ki67) and disease stage were derived from pre-treatment core biopsy and clinical staging (cTNM) for patients receiving neoadjuvant therapy, and from final surgical pathology assessment (pTNM) for those proceeding directly to surgery. Other variables included the date and age at diagnosis, and Nottingham histologic grade (I, II, or III).

ER percentage was stratified into five subgroups: ER^0%^ (ERzero), ER^1–9%^ (ERlow), ER^10–29%^ (ERmild), ER^30–59%^ (ERmod), and ER^60–100%^ (ERhigh). The 30% threshold was guided by a penalized smoothing spline analysis, which identified a natural inflection point in the relationship between ER percentage and overall survival at approximately 30% (Supplementary Figure S1). The 60% threshold was chosen to further stratify the ER-high positive population based on prior literature defining strongly ER-positive tumors.^9^, ^10^, ^11^

Treatment data encompassed chemotherapy (pre- and postoperative), endocrine therapy (pre- and postoperative), and radiotherapy. Preoperative treatment data were used for patient filtration, while postoperative treatments were included in the statistical analyses as categorical variables.

The primary endpoint was OS, defined as the time from diagnosis to death from any cause. Alive patients and those lost to follow-up were censored at the end of this study (December 31, 2023). In the neoadjuvant setting, a censoring rule was applied at 10 years due to a few reported events, making it less reliable to estimate survival risks. In addition, pathologic complete response (pCR) was defined as the absence of residual invasive carcinoma in the breast and axillary lymph nodes at surgical pathology after neoadjuvant therapy, based on a cross-check of pathology reports.

### Gene expression analysis

For a subset of patients (n = 4767; diagnosed September 2010–May 2018), publicly available RNA sequencing data (in Fragments Per Kilobase per Million [FPKM]) and clinical information were obtained from the SCAN-B cohort.^12^ This cohort represents a molecular subset of the main study population. Five ER subgroups were defined identically. The FPKM values were log_2_-transformed after de-duplicating genes by keeping the gene with the highest expression. The principal component analysis (PCA) was performed on gene expression data. PAM50 intrinsic subtypes and risk of recurrence (ROR) scores obtained by the “NCN” strategy were used in the current study.^13^

### Statistical methods

Patient and tumor characteristics were investigated for the full cohort. Comparisons across the five ER subgroups (ERzero, ERlow, ERmild, ERmod, and ERhigh) were conducted using Pearson's Chi-squared test or Fisher's Exact Test for categorical variables and Kruskal–Wallis rank sum test for continuous variables.

Overall survival was analyzed by ER subgroups using the Kaplan–Meier method, and between-group differences were assessed using the log-rank test. Hazard ratios (HRs) with 95% confidence intervals (CIs) were estimated using univariable and multivariable Cox proportional hazards (PH) regression models. Two multivariable models were constructed. Model 1: HRs across ER subgroups were adjusted for tumor stage and grade, age at diagnosis, year of diagnosis, as well as chemotherapy, endocrine therapy, and radiotherapy. Model 2: Patients were grouped by ER subgroups and combined adjuvant treatment types (chemotherapy alone [CTonly], endocrine therapy alone [ETonly], both [CT + ET], or neither [None]). In this model, HRs were stratified by radiotherapy due to non-proportional hazards and were adjusted for tumor stage and grade, age at diagnosis, and year of diagnosis.

The continuous relationship between ER percentage and survival was estimated using a penalized smoothing spline Cox model (4 freedom), adjusted for Model 1. To assess potential overfitting due to imbalanced subgroup sizes, we calculated events-per-variable (EPV) ratios and performed bootstrap validation (1000 resamples) for ERmild subgroup (ETonly vs. CT + ET; Model 2). To address potential confounding by indication, we performed inverse probability of treatment weighting (IPTW) as a sensitivity analysis. Propensity scores were estimated using multivariable logistic regression, including all covariates from Model 1 and 2. IPTW weights were then applied to the Cox proportional hazards models (Model 1 and 2) to obtain weighted HRs with 95% CIs.

Complete case analysis was performed; missing data were minimal for most covariates (≤3.7%) (Supplementary Table S1).

Analyses and visualizations were completed using R (v4.5.1). Analyses were performed by the survminer (v0.5.0), survival (v3.8), WeightIt (v1.6.0), spline (v4.5.1) and boot (v1.3-31) packages. Results were visualized with ggplot2 (v3.5.2) and forestplot (v3.1.7). Data were summarized by gtsummary (v2.3.0). All tests were two-sided, with a significant threshold of P < 0.05.

### Role of the funding source

This study received no external funding.

### Ethical approval

The study was approved by the Regional Ethical Review Committee in Stockholm, Sweden.

## Results

### Cohort characteristics

Patient and tumor characteristics of the full cohort (n = 75,211) are summarized in Table 1, stratified by ER percentage. The ER^60–100%^ (ERhigh) comprised the majority (87.8%), followed by ER^0%^ (ERzero; 8.4%), ER^30–59%^ (ERmod; 2.1%), ER^1–9%^ (ERlow; 1.0%), and ER^10–29%^ (ERmild; 0.8%).

**Table 1:** Patient- and tumor characteristics in a population-based cohort of 75,211 eligible women with breast cancer diagnosed between 2007 and 2023, by ER subgroups.

Patient/tumor characteristics by ER subgroups	ERzero	ERlow	ERmild	ERmod	ERhigh	P-value^b^
	N = 6291^a^	N = 737^a^	N = 630^a^	N = 1547^a^	N = 66,006^a^	
Age at diagnosis	62.0 (21.0, 99.0)	62.0 (25.0, 95.0)	60.0 (23.0, 95.0)	61.0 (23.0, 99.0)	65.0 (21.0, 102.0)	<0.001
Age at diagnosis (group)						<0.001
<40	594 (9.4%)	76 (10.3%)	67 (10.6%)	93 (6.0%)	1581 (2.4%)	
40–49	1006 (16.0%)	117 (15.9%)	136 (21.6%)	290 (18.7%)	8630 (13.1%)	
50–64	1911 (30.4%)	224 (30.4%)	175 (27.8%)	546 (35.3%)	22,274 (33.7%)	
65–79	1978 (31.4%)	259 (35.1%)	187 (29.7%)	476 (30.8%)	27,145 (41.1%)	
≥80	802 (12.7%)	61 (8.3%)	65 (10.3%)	142 (9.2%)	6376 (9.7%)	
Year of diagnosis (Period)						<0.001
2007–2012	953 (15.1%)	183 (24.8%)	195 (31.0%)	944 (61.0%)	14,937 (22.6%)	
2013–2016	1567 (24.9%)	194 (26.3%)	167 (26.5%)	295 (19.1%)	18,328 (27.8%)	
2017–2020	2236 (35.5%)	200 (27.1%)	146 (23.2%)	177 (11.4%)	18,399 (27.9%)	
2021–2023	1535 (24.4%)	160 (21.7%)	122 (19.4%)	131 (8.5%)	14,342 (21.7%)	
Progesterone receptor						<0.001
Negative (<10%)	6108 (97.1%)	668 (90.6%)	379 (60.2%)	404 (26.1%)	8445 (12.8%)	
Positive (≥10%)	175 (2.8%)	44 (6.0%)	205 (32.5%)	1054 (68.1%)	56,562 (85.7%)	
Unknown	8 (0.1%)	25 (3.4%)	46 (7.3%)	89 (5.8%)	999 (1.5%)	
Ki67 (median)	63.0 (0.0, 100.0)	55.5 (0.0, 100.0)	48.0 (1.0, 100.0)	28.0 (0.0, 100.0)	18.0 (0.0, 100.0)	<0.001
Unknown	623	153	158	830	11,694	
TNM stage (clin/path)						<0.001
0	4 (0.1%)	3 (0.4%)	0 (0.0%)	0 (0.0%)	105 (0.2%)	
I	2404 (38.2%)	258 (35.0%)	233 (37.0%)	749 (48.4%)	38,238 (57.9%)	
II	3247 (51.6%)	407 (55.2%)	323 (51.3%)	602 (38.9%)	22,568 (34.2%)	
III	602 (9.6%)	67 (9.1%)	69 (11.0%)	184 (11.9%)	4810 (7.3%)	
Unknown	34 (0.5%)	2 (0.3%)	5 (0.8%)	12 (0.8%)	285 (0.4%)	
Grade (NHG)						<0.001
I	113 (1.8%)	45 (6.1%)	45 (7.1%)	321 (20.7%)	16,197 (24.5%)	
II	1126 (17.9%)	176 (23.9%)	187 (29.7%)	694 (44.9%)	37,946 (57.5%)	
III	4075 (64.8%)	395 (53.6%)	315 (50.0%)	448 (29.0%)	10,331 (15.7%)	
Unknown	977 (15.5%)	121 (16.4%)	83 (13.2%)	84 (5.4%)	1532 (2.3%)	
Lymph node metastases						<0.001
1–3	780 (12.4%)	120 (16.3%)	96 (15.2%)	276 (17.8%)	13,268 (20.1%)	
None	3879 (61.7%)	429 (58.2%)	366 (58.1%)	916 (59.2%)	44,229 (67.0%)	
Not available	11 (0.2%)	0 (0.0%)	0 (0.0%)	3 (0.2%)	86 (0.1%)	
Unknown	1621 (25.8%)	188 (25.5%)	168 (26.7%)	352 (22.8%)	8423 (12.8%)	
Chemotherapy						<0.001
Adjuvant	2633 (41.9%)	277 (37.6%)	221 (35.1%)	413 (26.7%)	13,037 (19.8%)	
Neoadj and postneoadj	603 (9.6%)	63 (8.5%)	34 (5.4%)	29 (1.9%)	631 (1.0%)	
Neoadj only	950 (15.1%)	120 (16.3%)	85 (13.5%)	79 (5.1%)	1697 (2.6%)	
No	994 (15.8%)	136 (18.5%)	183 (29.0%)	733 (47.4%)	41,788 (63.3%)	
Unknown	1111 (17.7%)	141 (19.1%)	107 (17.0%)	293 (18.9%)	8853 (13.4%)	
Endocrine therapy						<0.001
Adjuvant	100 (1.6%)	39 (5.3%)	284 (45.1%)	1017 (65.7%)	48,087 (72.9%)	
Neoadj and postneoadj	10 (0.2%)	2 (0.3%)	6 (1.0%)	11 (0.7%)	682 (1.0%)	
Neoadj only	28 (0.4%)	1 (0.1%)	1 (0.2%)	1 (0.1%)	36 (0.1%)	
No	5215 (82.9%)	569 (77.2%)	239 (37.9%)	230 (14.9%)	8382 (12.7%)	
Unknown	938 (14.9%)	126 (17.1%)	100 (15.9%)	288 (18.6%)	8819 (13.4%)	
Combined adjuvant treatment						<0.001
CT + ET	94 (1.5%)	18 (2.4%)	172 (27.3%)	421 (27.2%)	13,522 (20.5%)	
ETonly	64 (1.0%)	40 (5.4%)	180 (28.6%)	703 (45.4%)	37,234 (56.4%)	
CTonly	3141 (49.9%)	321 (43.6%)	83 (13.2%)	21 (1.4%)	180 (0.3%)	
None	1880 (29.9%)	215 (29.2%)	88 (14.0%)	109 (7.0%)	6176 (9.4%)	
Unknown	1112 (17.7%)	143 (19.4%)	107 (17.0%)	293 (18.9%)	8894 (13.5%)	
Adjuvant radiotherapy						<0.001
Yes	4039 (64.2%)	456 (61.9%)	392 (62.2%)	916 (59.2%)	43,975 (66.6%)	
No	1146 (18.2%)	140 (19.0%)	133 (21.1%)	340 (22.0%)	13,158 (19.9%)	
Unknown	1106 (17.6%)	141 (19.1%)	105 (16.7%)	291 (18.8%)	8873 (13.4%)	
Screening detected						<0.001
Yes	1943 (30.9%)	264 (35.8%)	229 (36.3%)	726 (46.9%)	36,088 (54.7%)	
No	4333 (68.9%)	471 (63.9%)	401 (63.7%)	819 (52.9%)	29,802 (45.2%)	
Unknown	15 (0.2%)	2 (0.3%)	0 (0.0%)	2 (0.1%)	116 (0.2%)	

Clinical stage (cTNM) is shown for neoadjuvant patients; pathological stage (pTNM) is shown for adjuvant patients.

Abbreviations: ER subgroups, ER0% (ERzero), ER1–9% (ERlow), ER10–29% (ERmild), ER30–59% (ERmod), and ER60–100% (ERhigh). Combined adjuvant treatment: CT + ET, chemotherapy and endocrine therapy; ETonly, endocrine therapy alone; CTonly, chemotherapy alone; None, neither chemotherapy nor endocrine therapy.

Note: “Not available” in Lymph node metastases indicates patients who received neoadjuvant treatment and achieved a pathologic complete response.

a Median (Min, Max); n (%).

b Kruskal–Wallis rank sum test; Pearson's Chi-squared test with simulated P-value (based on 2000 replicates); Fisher's Exact Test for Count Data with simulated P-value (based on 2000 replicates).

As expected, treatment patterns varied substantially across ER subgroups, as suggested by international and national treatment guidelines. In the ERhigh, ERmod, and ERmild subgroups, the most common postoperative treatment (Combined adjuvant treatment) was endocrine therapy alone (ETonly: 56.4%, 45.4%, and 28.6%, respectively). Postoperative chemotherapy plus endocrine therapy (CT + ET) was the second most frequent approach in these three subgroups (20.5%, 27.2%, 27.3%, respectively). In the ERzero and ERlow subgroups, postoperative chemotherapy alone (CTonly) was the predominant treatment (49.9% and 43.6%, respectively). Accordingly, ETonly was the most common treatment (50.8%) in the full cohort, followed by CT + ET (18.9%) (Supplementary Table S1).

Year of diagnosis, a variable included in the multivariable model, was evenly distributed across four periods: 2007–2012 (22.9%), 2013–2016 (27.3%), 2017–2020 (28.1%), and 2021–2023 (21.7%) (Supplementary Figure S2 and Table S1). The majority of ER percentages fell into 90–100% bin, followed by 0–10% bin, across all diagnostic periods (Supplementary Figure S3). Tumor characteristics may reflect the underlying biology of the ER continuum. For Nottingham Histologic Grade (NHG), lower ER expression was associated with higher grade (Supplementary Figure S4). Grade III tumors predominated in the ERzero (76.7%), ERlow (64.1%) and ERmild (57.6%) subgroups, whereas grade II was most frequent in the ERmod (47.4%) and ERhigh (58.9%) subgroups. Consistent with this, Ki67 expression demonstrated an inverse relationship with ER expression, with median Ki67 decreasing progressively across higher ER subgroups (Supplementary Figure S5). Lymph nodes metastasis distribution was similar across ER subgroups, with 1–3 positive nodes in 12.4%–20.1% of patients (Table 1). The median follow-up was 7.01 years (interquartile range, 3.46–10.70 years) for the full cohort.

### Survival analysis by ER subgroups

**Fig. 2:**
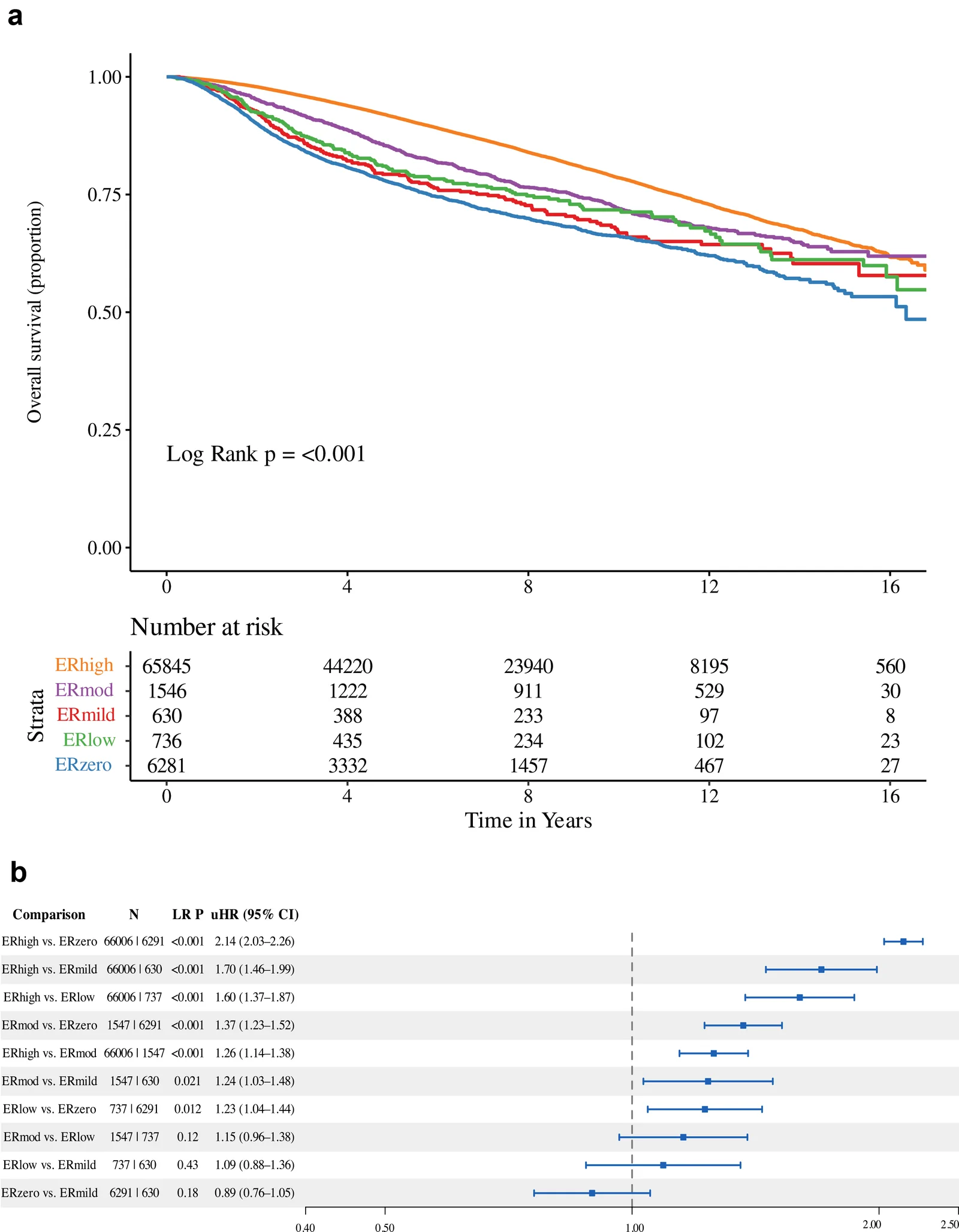
**Unadjusted overall survival by ER subgroups (n = 75,211)**. (a) Kaplan–Meier survival curves. (b) Forest plot of unadjusted hazard ratios (uHR). The reference group is listed first in each comparison. LR P, log-rank P value; uHR, unadjusted hazard ratios.

In the unadjusted analysis of the full cohort, overall survival (OS) showed a continuum of decreasing survival as ER percentage decreased (Fig. 2).

Using the ERhigh subgroup as the reference, survival was progressively and significantly worse with lower ER expression. The ERmod subgroup exhibited a 26% higher risk of death than the ERhigh subgroup (unadjusted hazard ratio [uHR] = 1.26, 95% confidence interval [CI]: 1.14–1.38, P < 0.001). This trend continued with the ERmild, which demonstrated the highest mortality risk among non-ERzero subgroups, with a 70% higher risk than both ERhigh (uHR = 1.70, 95% CI: 1.46–1.99, P < 0.001) and a 24% higher risk than ERmod (uHR = 1.24, 95% CI: 1.03–1.48, P = 0.021).

Patients with ERlow tumors had a 60% higher risk of death than those with ERhigh tumors (uHR = 1.60, 95% CI: 1.37–1.87, P < 0.001), but were similar to those with ERmod tumors (uHR = 1.15, 95% CI: 0.96–1.38, P = 0.12). Notably, the ERmild subgroup showed mortality risk comparable to either ERzero (uHR = 0.89, 95% CI: 0.76–1.05, P = 0.18) or ERlow (uHR = 1.09, 95% CI: 0.88–1.36, P = 0.43). As expected, ERzero patients had the highest mortality risk in comparisons: ERhigh vs. ERzero (uHR = 2.14, 95% CI: 2.03–2.26, P < 0.001), ERmod vs. ERzero (uHR = 1.37, 95% CI: 1.23–1.52, P < 0.001), and ERlow vs. ERzero (uHR = 1.23, 95% CI: 1.04–1.44, P = 0.012).

**Fig. 3:**
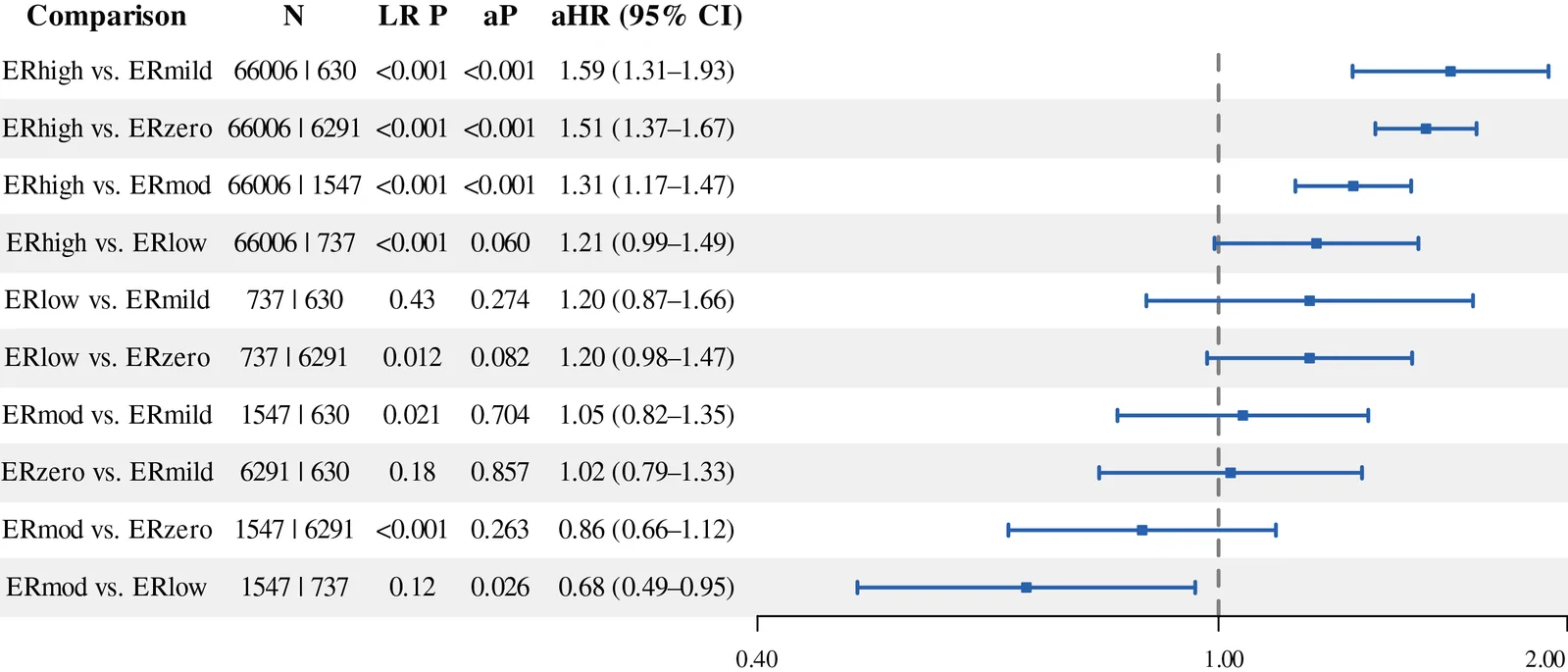
**Multivariable Cox regression analysis by ER subgroups**. Forest plot showing adjusted hazard ratios (aHR) for overall survival. The model was adjusted for tumor stage, grade, age, year of diagnosis, and postoperative therapy (Model 1). The reference group for each comparison is listed first. LR P, unadjusted log-rank P value; aP, adjusted P value; aHR, adjusted hazard ratios.

Following adjustment for tumor stage and grade, age, year of diagnosis, and postoperative therapy (Model 1), the poor survival outcome remained for the ERmild (adjusted HR [aHR] = 1.59, 95% CI: 1.31–1.93, adjusted P [aP] < 0.001) and ERmod (aHR = 1.31, 95% CI: 1.17–1.47, aP < 0.001), and ERzero (aHR = 1.51, 95% CI: 1.37–1.67, aP < 0.001) subgroups compared to the ERhigh subgroup (Fig. 3). After adjustment, ERlow showed a lower mortality risk compared to ERmod after multivariable adjustment (aHR = 0.68, 95% CI: 0.49–0.95, aP = 0.026).

Together, ER expression serves as a continuous prognostic biomarker. The ERmild (10–29%) subgroup demonstrates survival outcomes similar to those of ERlow and ERzero tumors, whereas ERmod (30–59%) tumors retain a significantly worse prognosis than ERhigh tumors, even after comprehensive adjustment.

### Survival analysis by ER subgroups and treatment type

**Fig. 4:**
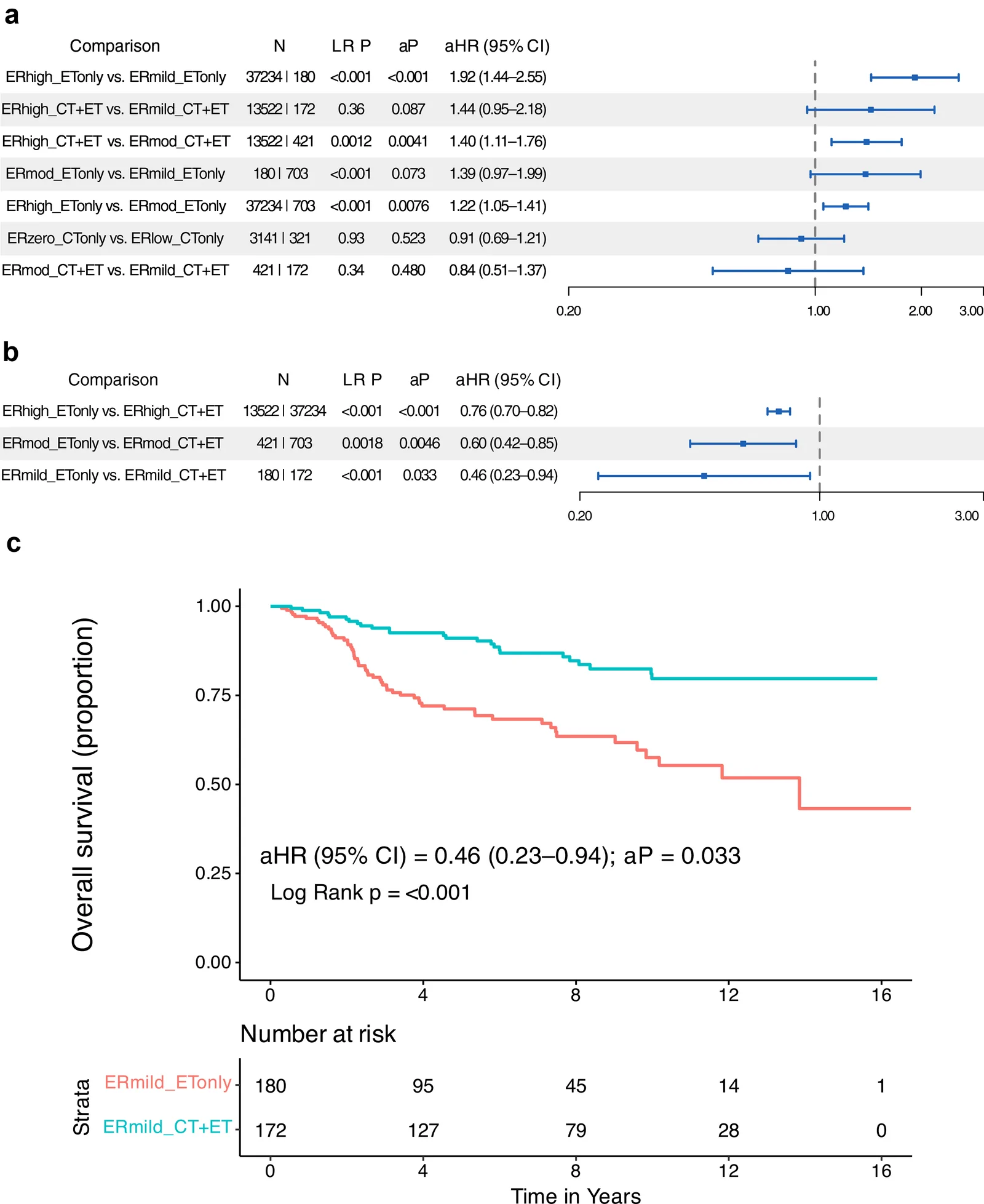
**Multivariable Cox regression analysis by ER subgroups and postoperative treatment**. (a) Forest plot by different ER subgroups with the same treatment: The model was adjusted for tumor stage, grade, age, diagnosis year, and stratified by radiotherapy (Model 2). (b) Forest plot of different treatments within the ER subgroup, showing adjusted hazard ratios (aHR) for overall survival from Model 2. Patients were grouped by ER subgroups and treatment type (CTonly, ETonly, CT + ET, None). The reference group for each comparison is listed as the first group. LR P, unadjusted log-rank P value; aP, adjusted P value; aHR, adjusted hazard ratios. (c) Representative Kaplan–Meier survival curve for a selected comparison (ERmild_ETonly vs. ERmild_CT + ET), with the corresponding aHR and aP from Model 2. Note: ERlow_CT + ET vs. ERlow_CTonly (in a), and ERlow_CT + ET vs. ERmod_CT + ET, ERlow_CT + ET vs. ERmild_CT + ET, ERhigh_CT + ET vs. ERlow_CT + ET (in b) were excluded because of extremely biased sample size.

We further investigate the combined impact of ER expression and treatment type on OS (Fig. 4).

For comparisons of ER subgroups within the same treatments, among patients receiving ETonly, those with ERmild tumors exhibited a 92% higher risk of death than those with ERhigh tumors (aHR = 1.92, 95% CI: 1.44–2.55, aP < 0.001). A similar, though less pronounced, risk was observed for ERhigh vs. ERmod patients on ETonly (aHR = 1.22, 95% CI: 1.05–1.41, aP < 0.0076). For patients treated with CT + ET, the ERmod subgroup demonstrated worse survival than the ERhigh subgroup (aHR = 1.40, 95% CI: 1.11–1.76, aP = 0.0041). In contrast, ERmod and ERmild patients showed similar mortality risks with both the ETonly and CT + ET categories.

When comparing treatment types within the same ER subgroup, the impact of adding chemotherapy varied dramatically by ER expression. For patients with ERmild tumors, CT + ET was associated with a 54% lower risk of death compared to the ETonly (aHR = 0.46, 95% CI: 0.23–0.94, aP = 0.033; Fig. 4c), though the confidence interval was wide, reflecting the limited sample size. Conversely, for patients with ERmod tumors, CT + ET was associated with a 40% lower risk compared to ETonly (aHR = 0.60, 95% CI: 0.42–0.85, aP = 0.0046). A similar trend, albeit of smaller magnitude, was observed in the ERhigh subgroup, where CT + ET was associated with a 24% lower risk than ETonly (aHR = 0.76, 95% CI: 0.70–0.82, aP < 0.001).

In summary, these analyses demonstrate: 1) Within the same treatments, patients with lower ER expressing tumors (ERmild and ERmod) had a worse prognosis compared to patients with ERhigh disease; and 2) The survival benefit of adding chemotherapy to endocrine therapy seems to be profound in the ERmild subgroup, where it halves the risk of death.

### Molecular characteristics by ER subgroups

**Fig. 5:**
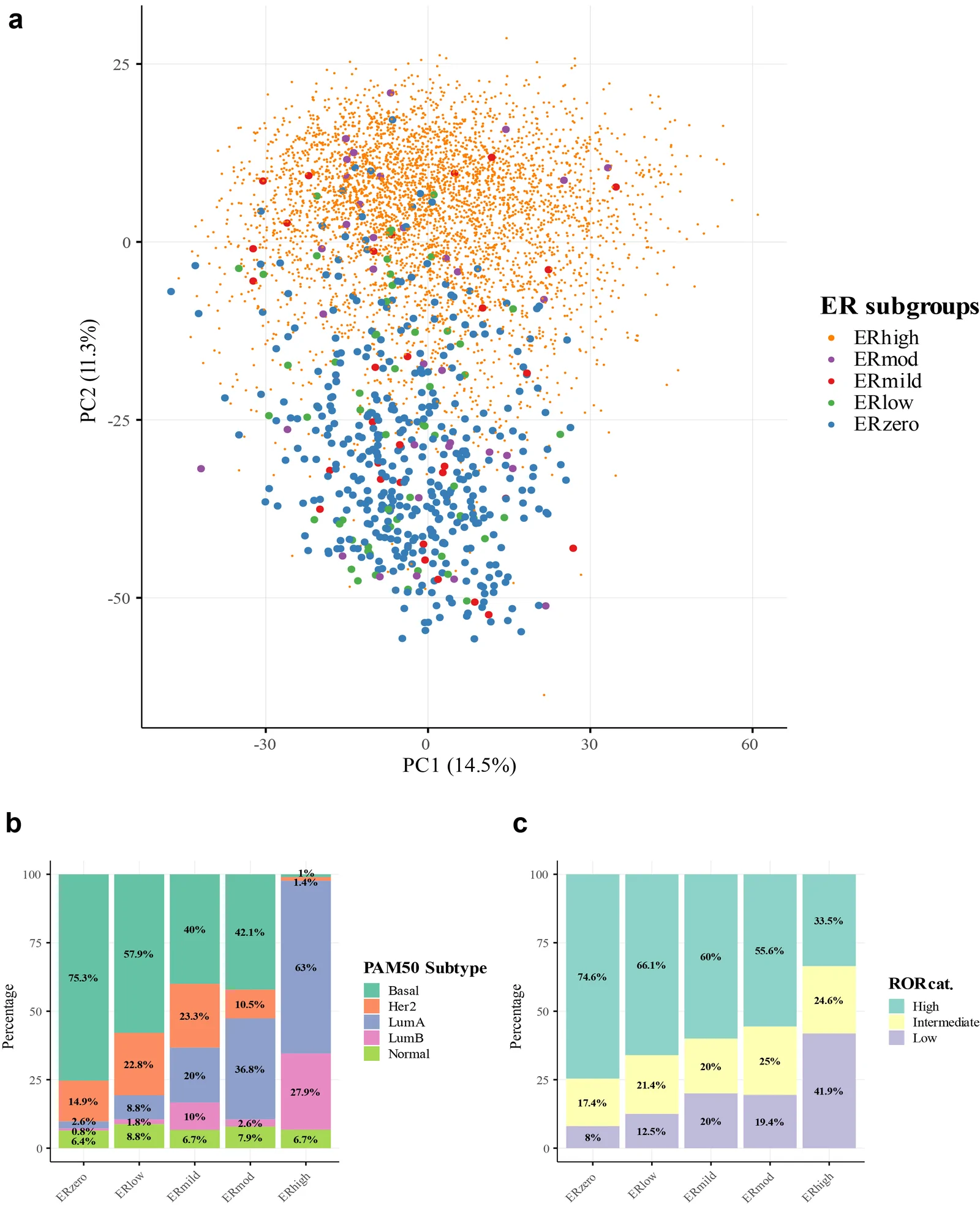
**Molecular characteristics by ER subgroups in SCAN-B (n = 4767)**. (a) Principal component analysis (PCA) plot showing molecular profiles. (b) Stacked bar chart showing the distribution of PAM50 intrinsic subtypes. (c) Stacked bar chart showing the distribution of risk of recurrence (ROR) score categories. ROR cat.: ROR category.

For a subset of patients, their gene expression data were available from SCAN-B. Clinical characteristics of these patients (n = 4767) are summarized in Supplementary Table S2. Principal component analysis (PCA) revealed molecularly distinct profiles for ER subgroups, with particularly homogeneous clustering of the ERzero, ERlow, ERmild, and ERmod subgroups, distinct from the ERhigh subgroup (Fig. 5a). The distribution of PAM50 intrinsic subtypes differed significantly across these subgroups (Fisher's exact test: P < 0.001, Fig. 5b). The basal-like subtype was dominant in ERzero, ERlow, ERmild, and ERmod subgroups, ranging from 40% to 53.5%, but constituted only 1.0% in the ERhigh subgroup. Conversely, the luminal A (LumA) subtype accounted for 63% of the ERhigh subgroup, with its prevalence declining sharply across groups, from 36.8% in ERmod subgroup to 2.6% in the ERzero subgroup. A similar trend was observed for the risk of recurrence (ROR) score, which also differed significantly across ER subgroups (Fisher's exact test: P < 0.001; Fig. 5c). The proportion of High ROR was lowest in the ERhigh subgroup and increased progressively to 74.6% in the ERzero subgroup. In contrast, the proportion of Low ROR scores in all non-ERhigh groups was more than 50% lower than that in the ERhigh subgroup.

### Sensitivity analysis

To assess the robustness of our findings, we performed several sensitivity analyses, including inverse probability of treatment weighting (IPTW), stratification by treatment setting (adjuvant and neoadjuvant), restricted to diagnostic periods, and bootstrap validation.

To address potential confounding by indication, we repeated the primary Cox regression using IPTW analysis. The IPTW-weighted results were consistent with the primary analysis: ER-mild (aHR = 1.60, 95% CI: 1.17–2.19) and ER-mod (aHR = 1.27, 95% CI: 1.11–1.45) subgroups remained at higher mortality risk than ERhigh (Supplementary Figure S6). The benefit of adding chemotherapy to endocrine therapy persisted for ERmild (ETonly vs. CT + ET, aHR = 0.21, 95% CI: 0.10–0.47, aP < 0.001) and ERmod (ETonly vs. CT + ET, aHR = 0.56, 95% CI: 0.37–0.86, aP = 0.0078) patients, while attenuated in ERhigh (Supplementary Figure S7).

Adding the detection method (screening vs. symptomatic) as a covariate did not alter our findings (Supplementary Figures S8 and S9). ER-mild continued to show worse survival than ER-high (aHR = 1.57, 95% CI: 1.29–1.91, aP < 0.001), and the benefit of CT + ET in ERmild remained significant (aHR = 0.46, 95% CI: 0.22–0.93, aP = 0.031).

Additional sensitivity analyses stratified by treatment setting (adjuvant vs. neoadjuvant) yielded similar findings (Supplementary Figures S10 and S11 and Tables S3 and S4). In the adjuvant setting (n = 70,159), the ERmod (aHR = 1.29, 95% CI: 1.15–1.45, aP < 0.001), ERmild (aHR = 1.94, 95% CI: 1.22–1.83, aP < 0.001), and ERzero (aHR = 1.38, 95% CI: 1.24–1.53, aP < 0.001) subgroups all showed worse survival than ERhigh subgroup. In contrast, the ERlow subgroup showed a 36% lower risk than the ERmod (aHR = 0.64, 95% CI: 0.45–0.92, aP = 0.014), indicating better survival with ERlow tumors compared to ERmod tumors in the adjuvant setting. In the neoadjuvant setting (n = 5036), the adjusted survival differences were attenuated and remained statistically significant only for the ERmild (aHR = 2.64, 95% CI: 1.32–5.29, aP = 0.0061) and ERzero (aHR = 2.26, 95% CI: 1.13–3.19, aP = 0.0036) vs. the ERhigh subgroup.

ER subgroup incidence stabilized from 2013 onward (Supplementary Table S5). Restricting the analysis to 2013–2023 confirmed the primary findings (Supplementary Figures S12 and S13). ERmild (aHR = 1.79, 95% CI: 1.40–2.29, aP < 0.001), ERzero (aHR = 1.74, 95% CI: 1.55–1.96, aP < 0.001), and ERhigh (aHR = 1.40, 95% CI: 1.13–1.74, aP = 0.0018) all had higher mortality than ERhigh. The benefit of CT + ET only persisted in ERhigh (aHR = 0.72, 95% CI: 0.66–0.79, aP < 0.001).

Event-per-variable (EPV) calculations for the ERmild and ERlow subgroups were 10.06 and 10.50 (Supplementary Table S6), respectively, meeting the recommended minimum of 10.^14^ EPV values for ERmild_CT + ET (EPV = 1.79) and ERmild_ETonly (EPV = 4.07) fell far below the ideal threshold (Supplementary Table S7), reflecting the real-world rarity of these treatment combinations. Bootstrap validation (1000 resamples) was performed for this comparison and yielded a bias-corrected HR of 0.46 (95% CI: 0.18–0.99), consistent with the primary estimate (aHR = 0.46, 95% CI: 0.23–0.94) (Supplementary Figure S14).

In summary, these sensitivity analyses support the robustness of our primary conclusion that the ERmild (10–29%) and ERmod (30–59%) subgroups exhibit inferior survival and that adding chemotherapy provides a treatment benefit in these subgroups.

### Prognostic value of pathologic complete response (pCR)

We further evaluated the prognostic impact of achieving a pathologic complete response (pCR) following neoadjuvant therapy across ER subgroups (Supplementary Figure S15 and Table S4). Across ER subgroups, the frequency of pCR differed markedly. The highest pCR rates were observed in patients with lower ER expression: 36.3% in ERzero, 32.9% in ERlow, and 24.6% in ERmild tumors, corresponding to 57,073 and 31 patients, respectively. pCR was less frequent in the ERmod and ERhigh subgroups.

The survival benefit associated with pCR was most unambiguous in the ERzero, ERlow, and ERmild subgroups. Patients with ERmild tumors who achieved pCR had an 88% lower risk of death compared to non-responders (uHR = 0.12, 95% CI: 0.02–0.86, P = 0.011). Significant benefits were also observed for ERzero (uHR = 0.22, 95% CI: 0.15–0.32, P < 0.001) and ERlow (uHR = 0.19, 95% CI: 0.07–0.53, P < 0.001). In contrast, achieving pCR was not associated with a statistically significant improvement in overall survival for patients with ERmod (uHR = 0.32, 95% CI: 0.08–1.31, P = 0.093) and ERhigh (uHR = 0.54, 95% CI: 0.26–1.14, P = 0.1) disease. We found that the prognostic benefit of pCR was most robust and statistically significant in tumors with ER expression below 30% (ERzero, ERlow, and ERmild). No significant heterogeneity was observed among ER subgroups in terms of improved overall survival after achieving a pCR, mainly due to limited event numbers in the ERmod and ERhigh subgroups (Supplementary Table S4).

## Discussion

Most studies have traditionally divided breast cancer (BC) into two groups: ER-positive and ER-negative. Consistently, Muftah et al. showed protein and gene expression patterns of BC tend to cluster in two distinct groups (<1% and >70%) rather than being distributed evenly and continuously.^9^ The more recently defined third category, ERlow (1–9%), has been uniformly recognized across guidelines. By contrast, ER-intermediate tumors encompass a broad, less standardized spectrum (10–90%), reflecting the lack of consensus in the literature. Only a limited number of studies have examined further subdivisions.^15^, ^16^, ^17^ Consequently, clinicopathological characteristics and prognostic outcomes of ER-intermediate tumors vary across studies, complicating direct comparisons.^18^

In our large, population-based cohort, most tumors (87.8%) were ER-strongly positive, while ERmild and ERmod tumors represented less than 1%–2.1% of cases (Table 1). The rarity of these groups makes it challenging to draw conclusions from population-based data regarding the optimal treatment strategies.^17^^,^^19^^,^^20^

In a study by Makhlouf et al., ER was analyzed as a continuous variable in their Cox regression model and was found to be an independent predictor of increased survival in multivariate analysis.^21^ In our study, the survival pattern suggests a prognostic threshold effect: patients with ER expression <60% tumors have a continuum of elevated risk compared to those with ERhigh tumors. These broadly similar unadjusted outcomes across the lower ER spectrum (0–59%) are mainly driven by shared adverse features, such as higher grade and stage. Yet the persistence of significant hazard ratios for ERmild and ERmod in adjusted models indicates that the level of ER expression within this intermediate range remains an independent prognostic factor, with decreasing ER conferring incrementally worse outcomes (Figs. 2 and 3). By contrast, a recent study with a smaller cohort showed better overall survival in the ER-intermediate group (11–70%) than in the ERlow group.^22^ Differences in ER cutoffs and population characteristics may partly explain the finding. Importantly, we found that ERhigh patients had better survival than ERmod patients within the same treatment groups, both with endocrine therapy alone and with combined endocrine therapy and chemotherapy (Fig. 4a). This suggests that the underlying tumor biology associated with high ER expression levels may be a determinant of prognosis.

Patients with ERmild and ERmod tumors who received endocrine therapy alone had worse survival than those treated with combined chemotherapy and endocrine therapy (Fig. 4b). A similar pattern was observed in the ERhigh group. This finding should be interpreted cautiously due to possible confounding factors, comorbidities, or patient preferences, but it may suggest benefits from combined treatment in these patient groups.

A recent population-based study showed that ERlow patients treated with neoadjuvant therapy who did not achieve pathological complete response had worse overall survival when adjuvant endocrine therapy was omitted.^19^ The benefit of adjuvant endocrine therapy could not be observed in patients who achieved pathological complete response or in those who received adjuvant chemotherapy in addition to endocrine therapy. It should be noted that Choong et al. included a heterogeneous population with PR- and HER2-positive tumors.^19^ PR-positive patients are likely to respond to endocrine therapy, whereas HER2-positive disease has a distinct biology. Taken together, this evidence suggests that patients with ERlow tumors have a subgroup, possibly driven by PR expression, that benefits from endocrine therapy.^23^ However, it is also possible that some ERlow tumors may actually represent the ERmild group, since the diagnosis was based on core biopsies that may not fully capture tumor heterogeneity, a well-recognized phenomenon.^24^ In practice, distinguishing between 1%, 10% and 20% ER expression is subjective, and pathologists are unlikely to separate these groups with high precision. Especially in the neoadjuvant treatment setting, rigidly applying a single ER percentage cut-off to limited biopsy samples can be challenging, and the concept of a cutoff oversimplifies the range of the full ER expression spectrum.

Zhang et al. reported that BC with 11–70% ER expression may constitute a separate subgroup in terms of clinicopathological characteristics.^11^ Matsumoto et al. similarly defined the ER-intermediate group as having ER expression between 10% and 2/3 and found that this group had worse survival characteristics than the ER-positive group, more closely resembling the ERlow and ERzero tumors in their cohort.^10^ They observed that improvements in survival over three decades have primarily benefited the ER > 2/3 group, whereas no such improvement in prognosis was seen in the ER-intermediate group. This pattern may reflect a higher prevalence of basal-like biology among the ER-intermediates, generally associated with reduced endocrine sensitivity. In contrast, our data suggest that the ERmild BC benefit from adding chemotherapy to endocrine therapy, as those receiving endocrine therapy alone had worse survival than those receiving combined therapy. However, the subgroup definitions are not identical across studies.

As ER-expression subgroups remain disputed, Voorwerk et al. stratified ER expression into five categories. They found that the immune microenvironment of breast tumors with ER 1–9% and ER 10–50% was similar to that of ER 0% tumors.^15^ Based on these findings, they highlighted the need to define subgroups that may benefit from immune checkpoint inhibition. These results may suggest that treatment opportunities for the patients in the ER-intermediate spectrum may be broader than traditionally recognized, warranting further research.^7^ In the subset of the SCAN-B cohort of our population-based study, the molecular profiles of ERzero, ERlow, ERmild and ERmod cases were distributed homogenously, while ERhigh tumors tended to form a more distinct cluster (Fig. 5). The distribution of basal-like intrinsic subtype and high ROR-category followed a gradual decrease from ERzero to ERhigh patient group.

The strength of our study lies in its population-based cohort with a long follow-up time. It sheds light on a less studied group in the literature using real-world data. However, a limitation of our registry-based study concerns the lack of central ER histopathological reviewing.^25^ Therefore, some discrepancies in the assessment are expected among the different centers in local evaluations, especially in patients with heterogeneous ER expression, and in general over time. Furthermore, treatments were not assigned through randomization but based on clinical judgment and patient characteristics, which might introduce selection bias. Furthermore, new neoadjuvant treatment therapy options, such as immunotherapy, were introduced during the study period, which may have influenced the observed outcomes. Finally, breast cancer-specific survival data were not available for this cohort; therefore, analyses were restricted to overall survival.

In conclusion, our findings support the growing evidence that ER expression appears to be a spectrum rather than a binary feature, and that ER levels correlate with distinct molecular profiles. Although clinical decision making relies on pragmatic cutoffs, reporting ER as a percentage can provide additional biological insight and partially overlapping biological groupings may better reflect tumor behavior. Our findings highlight the importance of careful assessment of ERlow, mild and moderate tumors (1–59%). Further research is needed to determine how these findings could be translated into clinical practice.

## Contributors

BA and JH conceptualized the study. All authors contributed to the study design. BA supervised the study and was responsible for data acquisition. QY, BA, IF, CB, and XC curated and performed the data quality control. QY performed the statistical analysis. All authors contribute to data interpretation. QY and CB wrote the original draft. All authors reviewed, edited, and approved the final manuscript.

## Data sharing statement

Due to Swedish and European data protection laws, the individual-level data supporting this study are not publicly available to protect patient confidentiality. Proposals for data access can be submitted to the Swedish National Quality Register for Breast Cancer (NKBC). Access is granted to qualified researchers subject to a data use agreement and requisite ethical approvals.

## Declaration of interests

BA has obtained speaker's honoraria from Pfizer. JH has received speaker's honoraria or advisory board remuneration from Roche, Novartis, AstraZeneca, Pfizer, Eli Lilly, MSD, Sakura, and Gilead, and has received institutional research support from Cepheid, Novartis, Roche, MSD, and AstraZeneca outside the current work. JH is a Swedish Association for Pathology board member. He is also a co-founder and shareholder of Stratipath AB. IZ has received honoraria from Novartis (institutional) and from BioMed Central (part of Springer Nature Group) for editorial work, unrelated to the current work. He also received an institutional research grant from Gilend outside the current work. IF is chairperson of the Swedish National Quality Register for Breast Cancer. IF has received institutional research grants from MSD for research projects performed under the Master Collaboration Agreement between MSD and Karolinska Institutet, unrelated to this work. XC received an institutional research grant from MSD outside the current work. QY received a travel grant from Radiumhemmet funds to present a poster of this work. CB has no disclosures to declare.
