# Supplementary material for: Patient characteristics, treatment patterns, and survival across estrogen receptor expression subgroups in HER2-negative breast cancer: a population-based cohort study
Source: Lancet Reg Health Eur. 2026 Aug 26;68:101826. doi: 10.1016/j.lanepe.2026.101826 (PMC13559916; doi:10.1016/j.lanepe.2026.101826)
Supplement: Supplementary file 1 [file mmc1.pdf]

Patient characteristics, treatment patterns, and survival across estrogen receptor expression subgroups in HER2-negative breast cancer: a population-based cohort study

Qiao Yang[1*], Ceren Boyaci[1,2*], Ioannis Zerdes[1,3], Irma Fredriksson[3-5], Xinsong Chen[1], Johan Hartman[1,2#] and Balazs Acs[1,2#]

[1]Department of Oncology and Pathology, Karolinska Institutet, Stockholm, Sweden.
[2]Department of Clinical Pathology and Cancer Diagnostics, Karolinska University Hospital, Stockholm, Sweden.
[3]Theme Cancer, Karolinska University Hospital and Comprehensive Cancer Center, Stockholm, Sweden.
[4]Department of Molecular Medicine and Surgery, Karolinska Institutet, Stockholm, Sweden.
[5]Department of Breast-, Endocrine Tumors and Sarcoma, Karolinska University Hospital, Stockholm, Sweden.
[*]Shared first authorship – equal contributions.
[#]Shared last authorship – equal contributions.

Corresponding author:

Balázs Ács

Email: balazs.acs@ki.se; Phone: +46 702289125

Department of Oncology and Pathology, Karolinska Institutet, Stockholm, Sweden

Department of Clinical Pathology and Cancer Diagnostics, Karolinska University Hospital, Stockholm, Sweden

Supplementary Figures:

Figure S1 Penalized smoothing spline analysis of the relationship between ER percentage and overall survival. 3
Figure S2 The distribution of eligible patients by year of diagnosis. 4
Figure S3 Distribution of ER percentages by diagnostic period. 5
Figure S4 Distribution of Nottingham Histologic Grade (NHG) across ER subgroups in the full cohort. 6
Figure S5 Box plots showing Ki67 expression levels across ER subgroups. 7
Figure S6 Inverse Probability of Treatment Weighting (IPTW) analysis by ER subgroups in the full cohort (n = 75,112). 8
Figure S7 Inverse Probability of Treatment Weighting (IPTW) analysis by ER subgroups and postoperative treatment in the full cohort. 9
Figure S8 Multivariable analysis by ER subgroups, adding the screen program to covariates (n = 75,211). 10
Figure S9 Multivariable analysis across ER subgroups by postoperative treatment, adding the screen program to covariates (n = 75,211). 11
Figure S10 Overall survival analysis by ER subgroups in adjuvant setting (n = 70,156). 12
Figure S11 Overall survival analysis by ER subgroups in the neoadjuvant setting (n = 5,055). 13
Figure S12 Multivariable analysis by ER subgroups, restricted to 2013–2023 (n = 57,871). 15
Figure S13 Multivariable analysis across ER subgroups by postoperative treatment, restricted to 2013–2023 (n = 57,871). 16
Figure S14 Bootstrap distribution of hazard ratios (ETonly vs. CT+ET) in ERmild tumors (n = 1000 resamples). ... 17
Figure S15 Impact of achieving a Pathologic Complete Response (pCR) on overall survival across ER subgroups following neoadjuvant therapy (n = 5055). 18

Supplementary Tables:

Table S1 Distribution of patient and tumor characteristics in the full cohort (n = 75,112). 19
Table S2 Distribution of cohort and tumor characteristics in the SCAN-B subset (n = 4767). 22
Table S3 Distribution of patient and tumor characteristics in the adjuvant setting by ER subgroups (n = 70,156). ... 23
Table S4 Distribution of patient and tumor characteristics in the neoadjuvant setting by ER subgroups (n = 5055). 25
Table S5 The distributions of the incidence of five ER subgroups by diagnostic period. 28
Table S6 The assessment of events per variable (EPV) across five ER subgroups. 29
Table S7 The assessment of events per variable (EPV) across five ER subgroups by postoperative treatment. 30

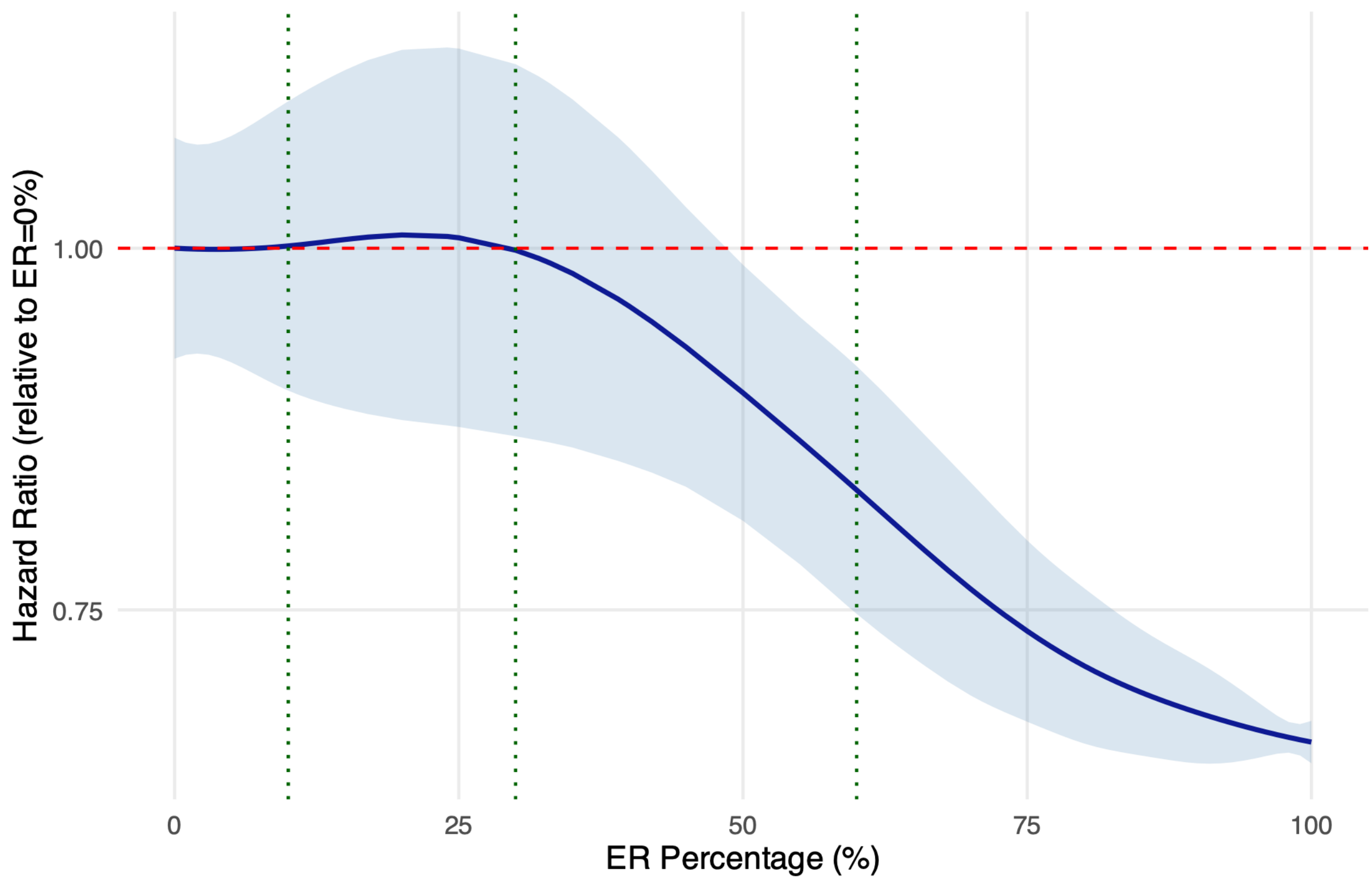

Figure S1 Penalized smoothing spline analysis of the relationship between ER percentage and overall survival.

The curve shows the hazard ratio for overall survival as a function of ER percentage, modeled as a continuous variable using a penalized smoothing spline, adjusted for age, tumor stage, grade, year of diagnosis, and postoperative treatment (Model 1). The reference is ER = 0%. Vertical dashed lines represent 10%, 30%, and 60%, respectively.

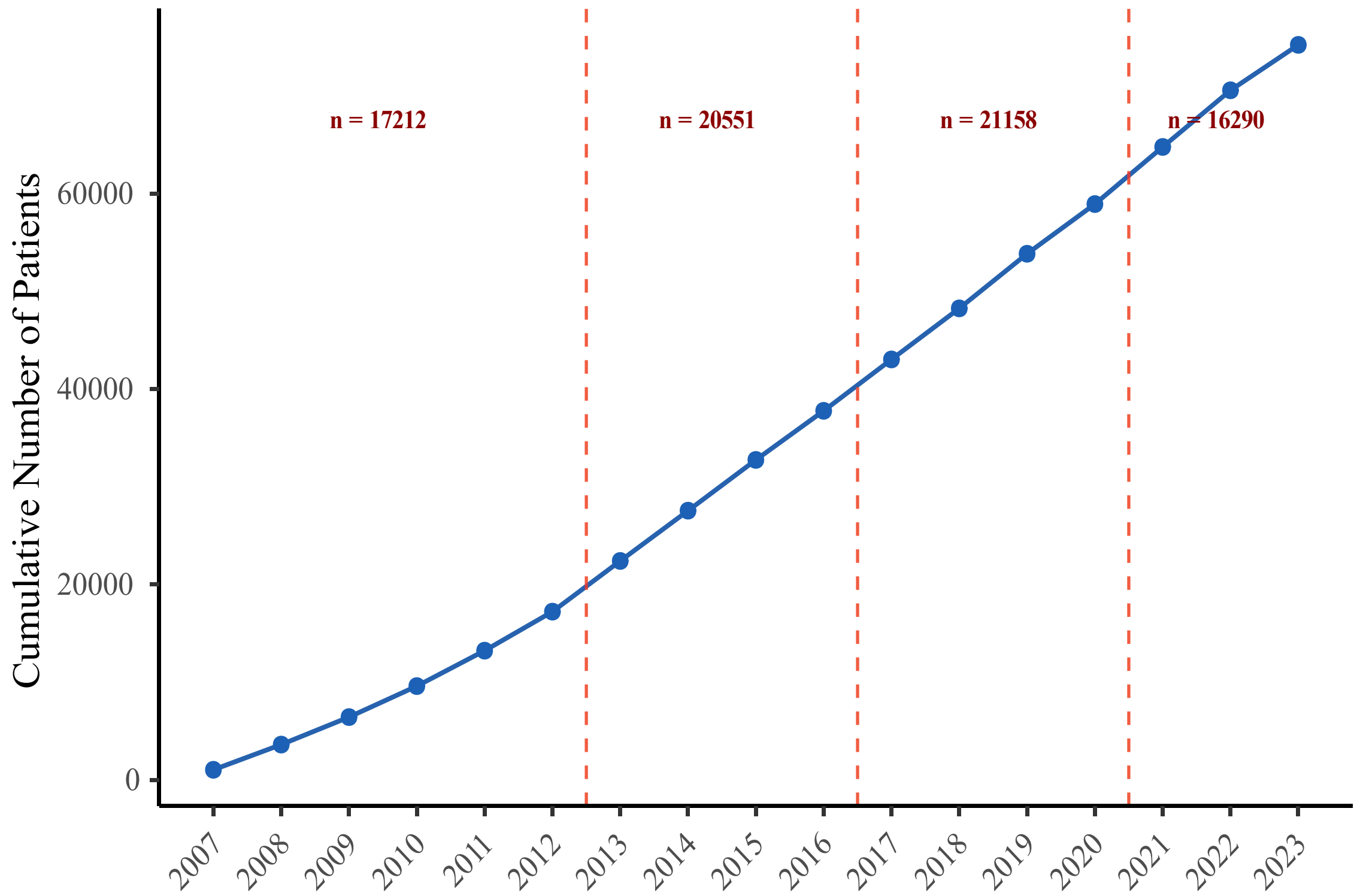

Figure S2 The distribution of eligible patients by year of diagnosis.

A cumulative line plot showing the number of patients included in the full cohort by year of diagnosis.

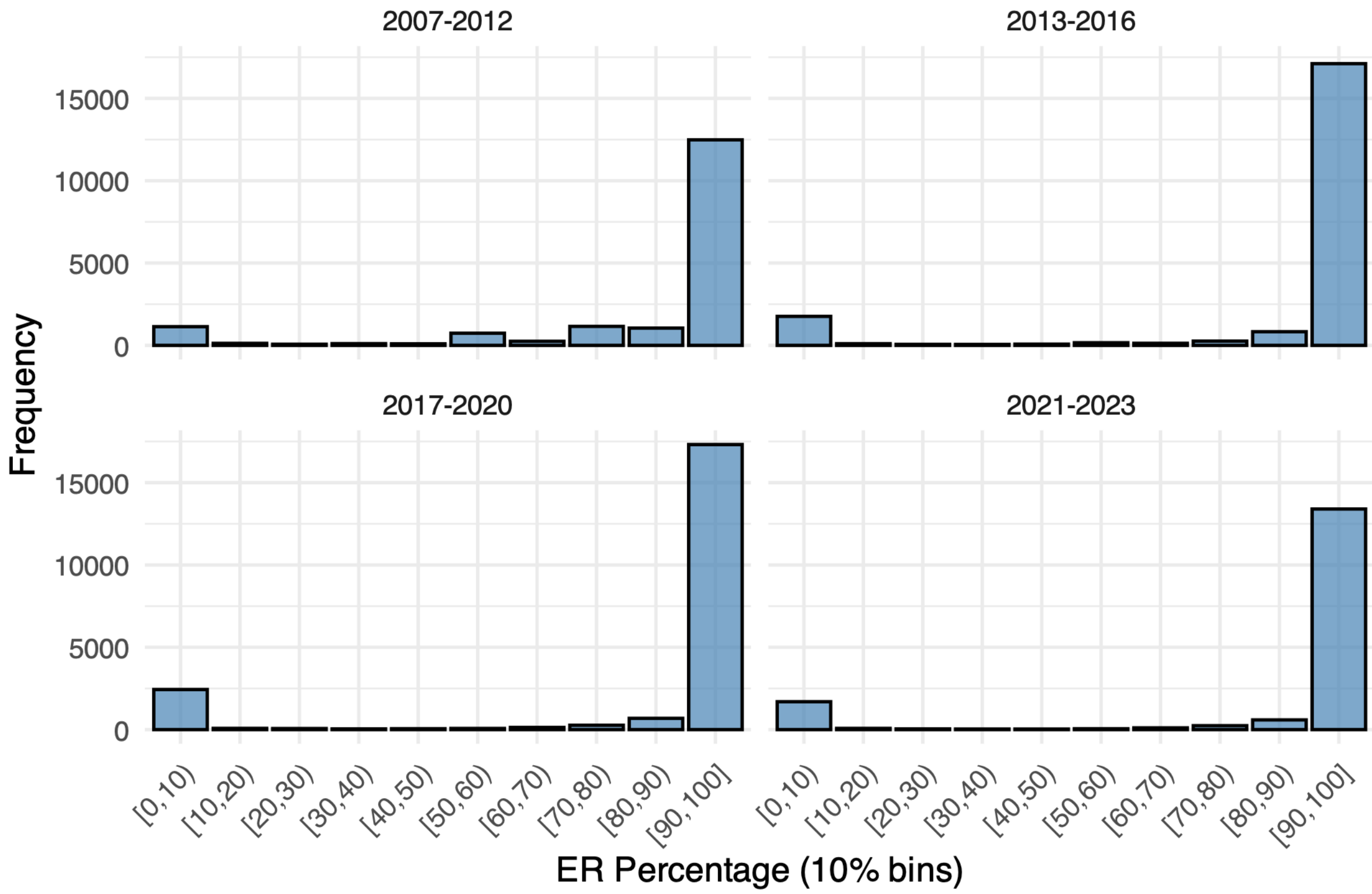

Figure S3 Distribution of ER percentages by diagnostic period.

The bar chart shows the frequency of cases within each 10% ER percentage bin, stratified by diagnostic period.

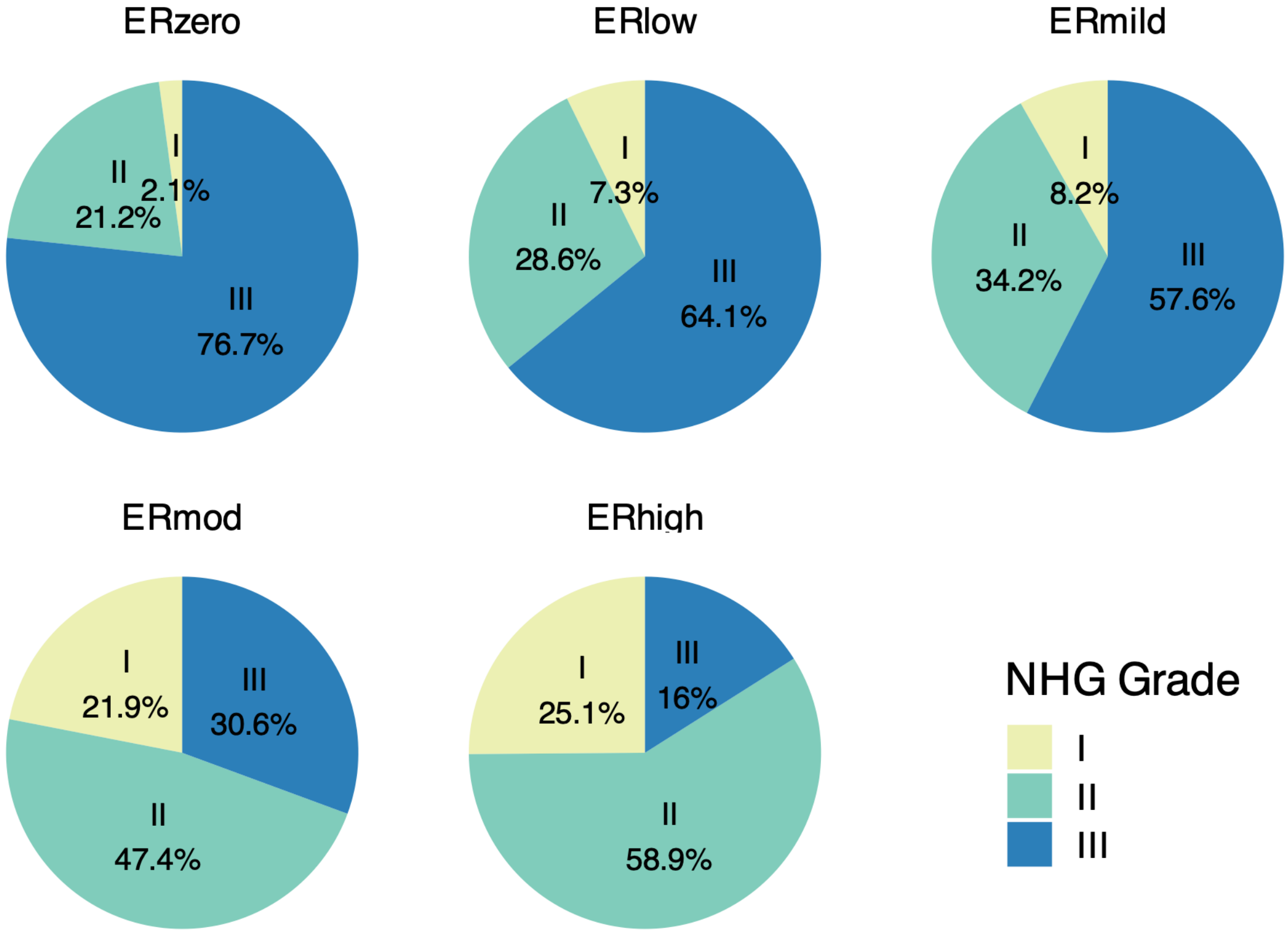

Figure S4 Distribution of Nottingham Histologic Grade (NHG) across ER subgroups in the full cohort.

ER subgroups are $ER^{0\%}$ (ERzero), $ER^{1\text{-}9\%}$ (ERlow), $ER^{10\text{-}29\%}$ (ERmild), $ER^{30\text{-}59\%}$ (ERmod), and $ER^{60\text{-}100\%}$ (ERhigh).

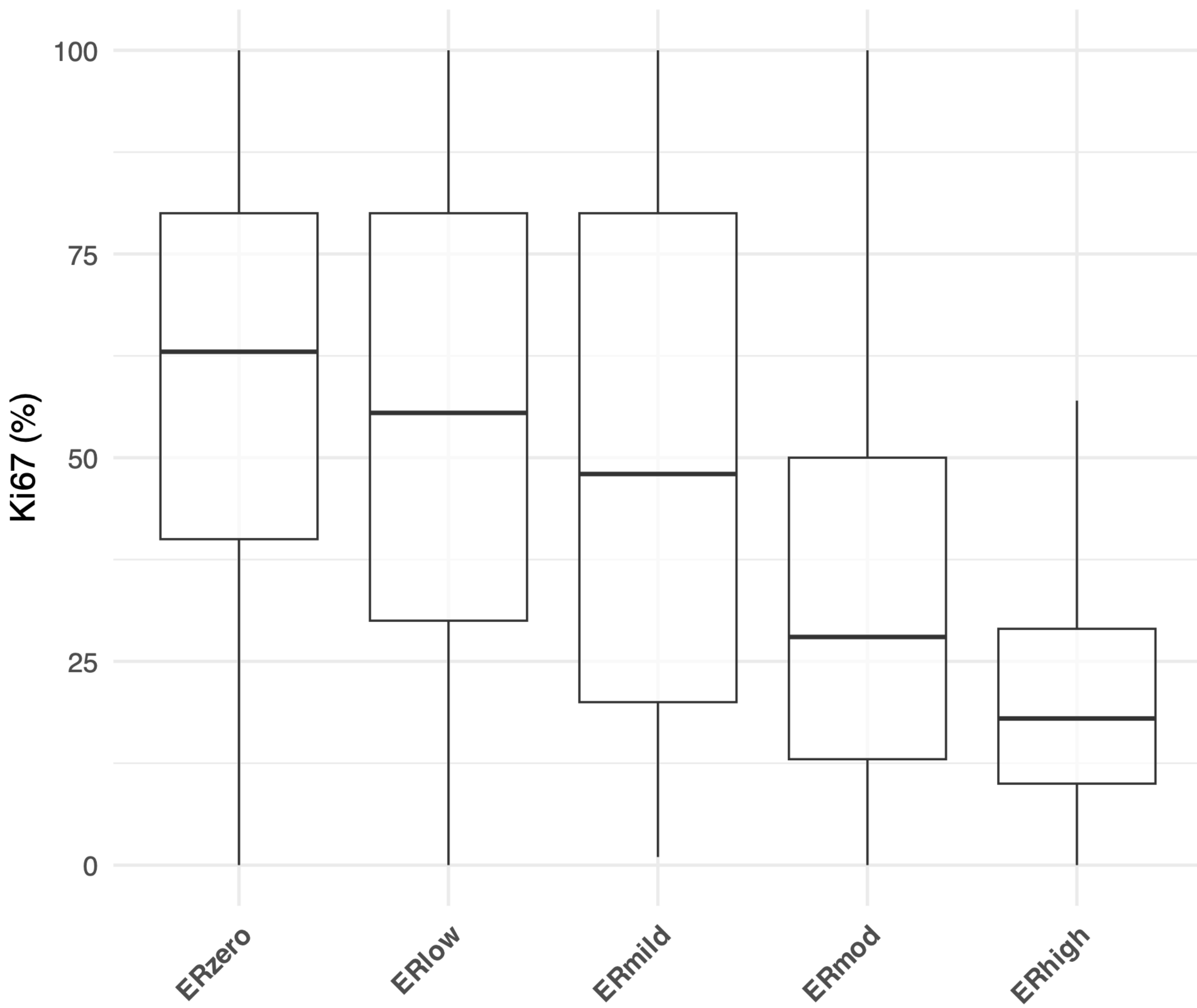

Figure S5 Box plots showing Ki67 expression levels across ER subgroups.

Comparison	N	aP	aHR (95% CI)
ERhigh vs. ERlow	66006 | 737	0.027	2.38 (1.11–5.13)
ERhigh vs. ERmild	66006 | 630	0.0035	1.60 (1.17–2.19)
ERhigh vs. ERmod	66006 | 1547	<0.001	1.27 (1.11–1.45)
ERmod vs. ERlow	1547 | 737	0.272	1.26 (0.83–1.92)
ERlow vs. ERzero	737 | 6291	0.124	1.15 (0.96–1.38)
ERzero vs. ERmild	6291 | 630	0.480	1.10 (0.84–1.45)
ERlow vs. ERmild	737 | 630	0.527	1.09 (0.83–1.43)
ERmod vs. ERmild	1547 | 630	0.607	1.06 (0.85–1.31)
ERmod vs. ERzero	1547 | 6291	0.281	0.77 (0.49–1.23)
ERhigh vs. ERzero	66006 | 6291	0.424	0.60 (0.17–2.08)

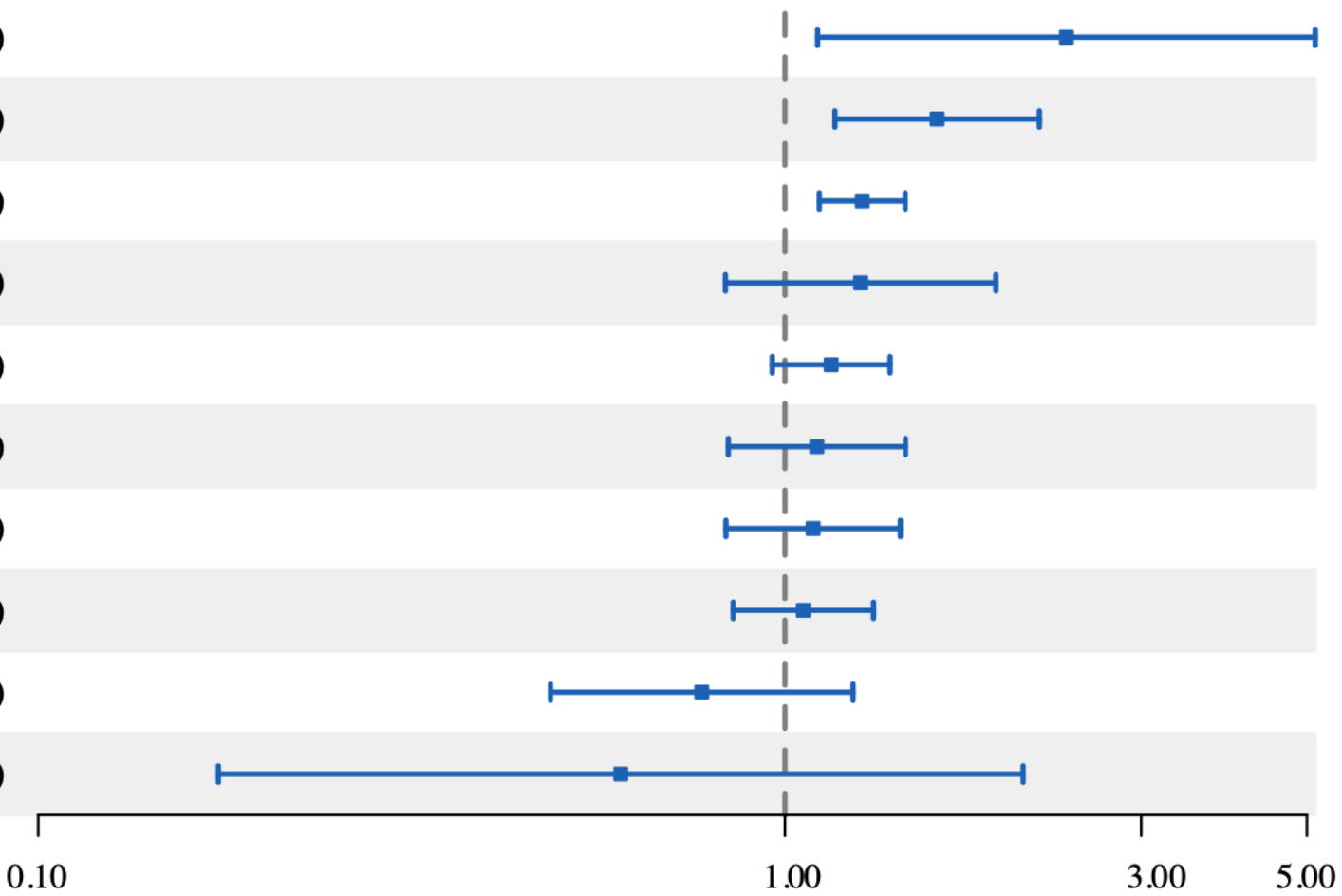

Figure S6 Inverse Probability of Treatment Weighting (IPTW) analysis by ER subgroups in the full cohort (n = 75,112).

Forest plot showing adjusted hazard ratios (aHR) with 95% confidence intervals (95% CI) from IPTW-weighted Cox proportional hazards models. Weights were estimated using propensity scores based on model 1, including tumor stage, grade, age at diagnosis, year of diagnosis, and postoperative therapy. The first group listed in each comparison serves as the reference. Abbreviations: aHR, adjusted hazard ratio; 95% CI, 95% confidence interval; IPTW, Inverse Probability of Treatment Weighting.

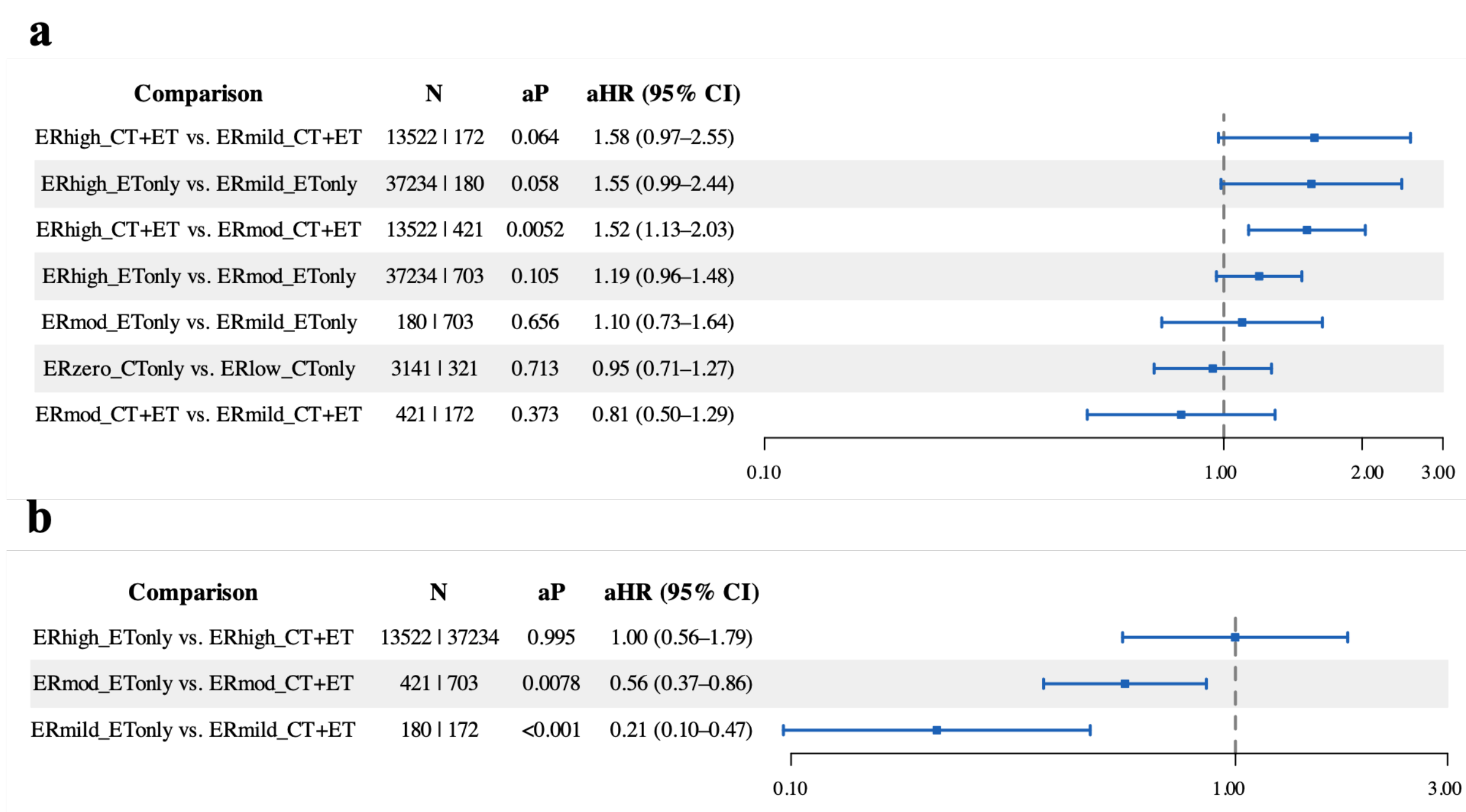

Figure S7 Inverse Probability of Treatment Weighting (IPTW) analysis by ER subgroups and postoperative treatment in the full cohort.

Forest plot showing adjusted hazard ratios (aHR) with 95% confidence intervals (95% CI) from IPTW-weighted Cox proportional hazards models. Weights were estimated using propensity scores based on model 2 (tumor stage, grade, age, and diagnosis year), stratified by radiotherapy. The first group listed in each comparison is the reference. (a) IPTW-weighted comparisons of ER subgroups within the same treatment type. (b) IPTW-weighted comparisons of treatment types within the same ER subgroups. Abbreviations: aP, adjusted p-value; aHR, adjusted hazard ratios. Note: ERlow_CT+ET vs. ERlow_CTonly (in a), and ERlow_CT+ET vs. ERmod_CT+ET, ERlow_CT+ET vs. ERmild_CT+ET, ERhigh_CT+ET vs. ERlow_CT+ET (in b) were excluded because of extremely biased sample size.

Comparison	N	LR P	aP	aHR (95% CI)
ERhigh vs. ERmild	66006 | 630	<0.001	<0.001	1.57 (1.29–1.91)
ERhigh vs. ERzero	66006 | 6291	<0.001	<0.001	1.45 (1.31–1.60)
ERhigh vs. ERmod	66006 | 1547	<0.001	<0.001	1.32 (1.17–1.48)
ERlow vs. ERmild	737 | 630	0.43	0.230	1.22 (0.88–1.68)
ERlow vs. ERzero	737 | 6291	0.012	0.115	1.18 (0.96–1.45)
ERhigh vs. ERlow	66006 | 737	<0.001	0.138	1.17 (0.95–1.43)
ERmod vs. ERmild	1547 | 630	0.021	0.724	1.05 (0.81–1.34)
ERzero vs. ERmild	6291 | 630	0.18	0.762	1.04 (0.80–1.35)
ERmod vs. ERzero	1547 | 6291	<0.001	0.155	0.82 (0.63–1.08)
ERmod vs. ERlow	1547 | 737	0.12	0.011	0.65 (0.46–0.91)

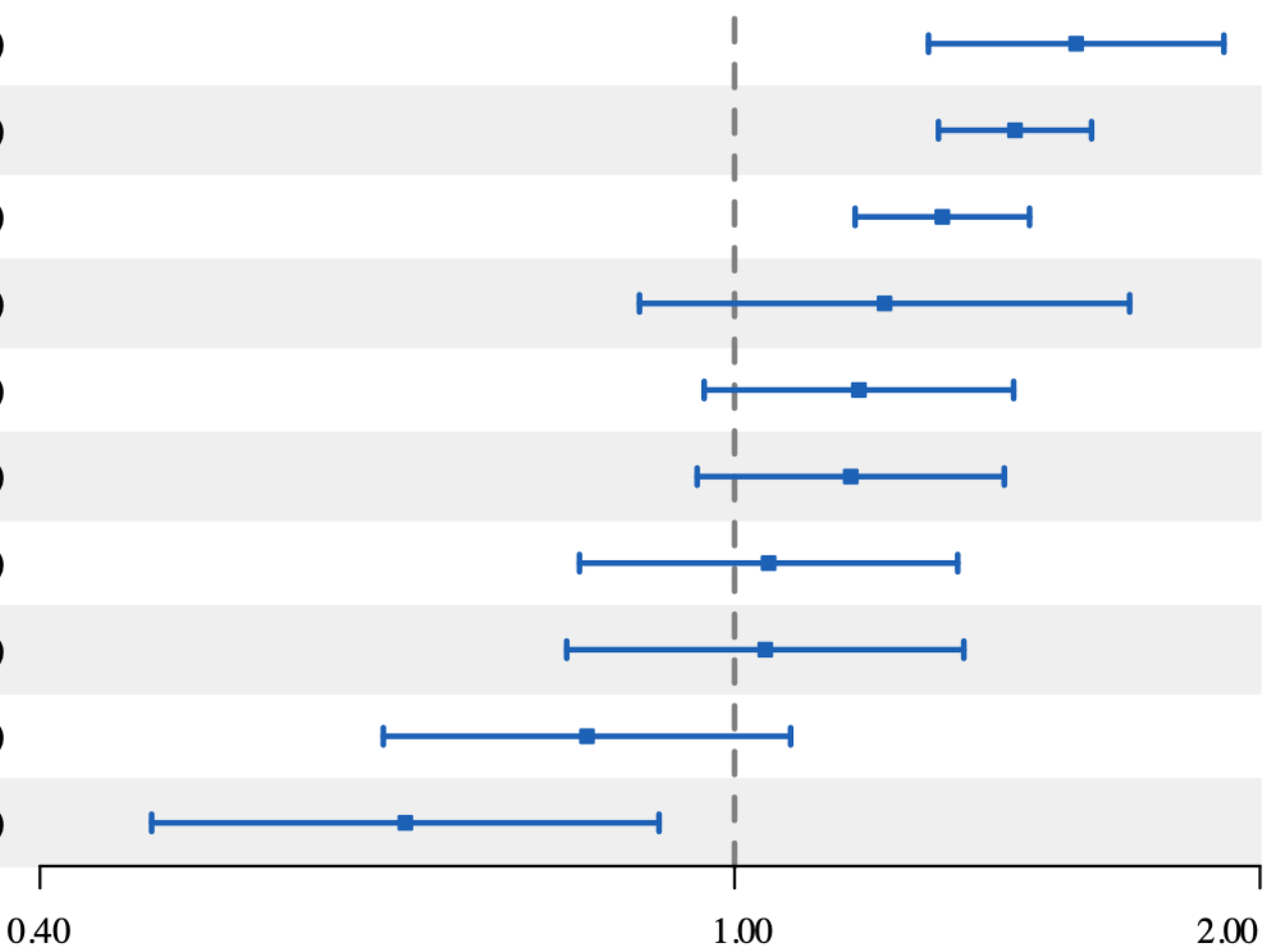

Figure S8 Multivariable analysis by ER subgroups, adding the screen program to covariates (n = 75,211).

Forest plot showing adjusted hazard ratios (aHR) with 95% confidence intervals (95% CI) from the Cox proportional hazards model (Model 1). The first group listed in each comparison serves as the reference. Abbreviations: aHR, adjusted hazard ratio; 95% CI, 95% confidence interval.

a

Comparison	N	LR P	aP	aHR (95% CI)
ERhigh_ETonly vs. ERmild_ETonly	37234 | 180	<0.001	<0.001	1.97 (1.48–2.62)
ERmod_ETonly vs. ERmild_ETonly	180 | 703	<0.001	0.050	1.44 (1.00–2.07)
ERhigh_CT+ET vs. ERmild_CT+ET	13522 | 172	0.36	0.111	1.40 (0.93–2.13)
ERhigh_CT+ET vs. ERmod_CT+ET	13522 | 421	0.0012	0.0038	1.40 (1.12–1.76)
ERhigh_ETonly vs. ERmod_ETonly	37234 | 703	<0.001	0.0061	1.23 (1.06–1.42)
ERzero_CTonly vs. ERlow_CTonly	3141 | 321	0.93	0.622	0.93 (0.70–1.23)
ERmod_CT+ET vs. ERmild_CT+ET	421 | 172	0.34	0.460	0.83 (0.51–1.36)

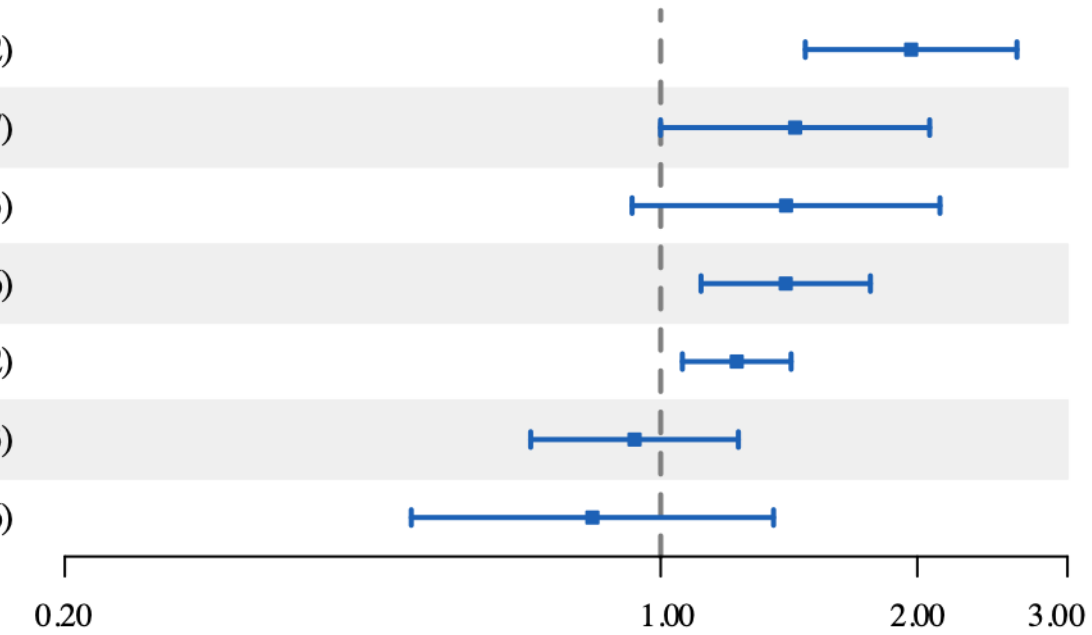

b

Comparison	N	LR P	aP	aHR (95% CI)
ERhigh_ETonly vs. ERhigh_CT+ET	13522 | 37234	<0.001	<0.001	0.76 (0.70–0.82)
ERmod_ETonly vs. ERmod_CT+ET	421 | 703	0.0018	0.0072	0.62 (0.43–0.88)
ERmild_ETonly vs. ERmild_CT+ET	180 | 172	<0.001	0.031	0.46 (0.22–0.93)

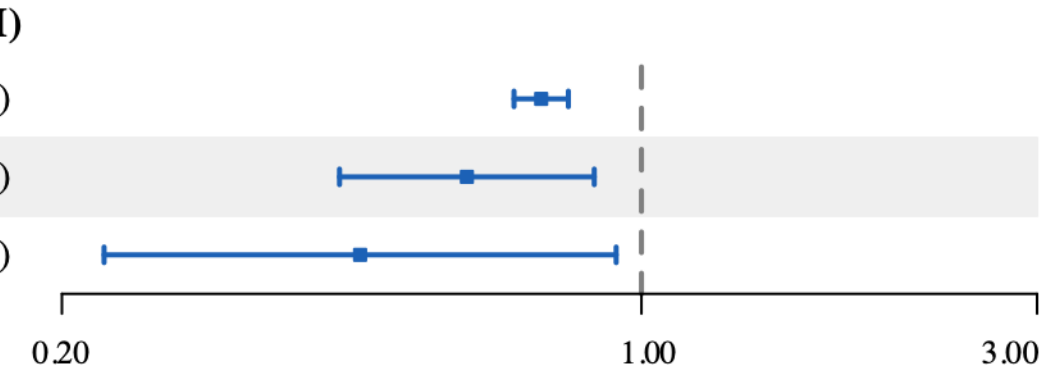

Figure S9 Multivariable analysis across ER subgroups by postoperative treatment, adding the screen program to covariates (n = 75,211).

(a) Forest plot by different ER subgroups with the same treatment: The model was adjusted for tumor stage, grade, age, diagnosis year, and stratified by radiotherapy (Model 2). (b) Forest plot of different treatments within the ER subgroup, showing adjusted hazard ratios (aHR) for overall survival from Model 2. LR P, unadjusted log-rank p value; aP, adjusted p value; aHR, adjusted hazard ratios. Note: ERlow_CT+ET vs. ERlow_CTonly (in a), and ERlow_CT+ET vs. ERmod_CT+ET, ERlow_CT+ET vs. ERmild_CT+ET, ERhigh_CT+ET vs. ERlow_CT+ET (in b) were excluded because of extremely biased sample size.

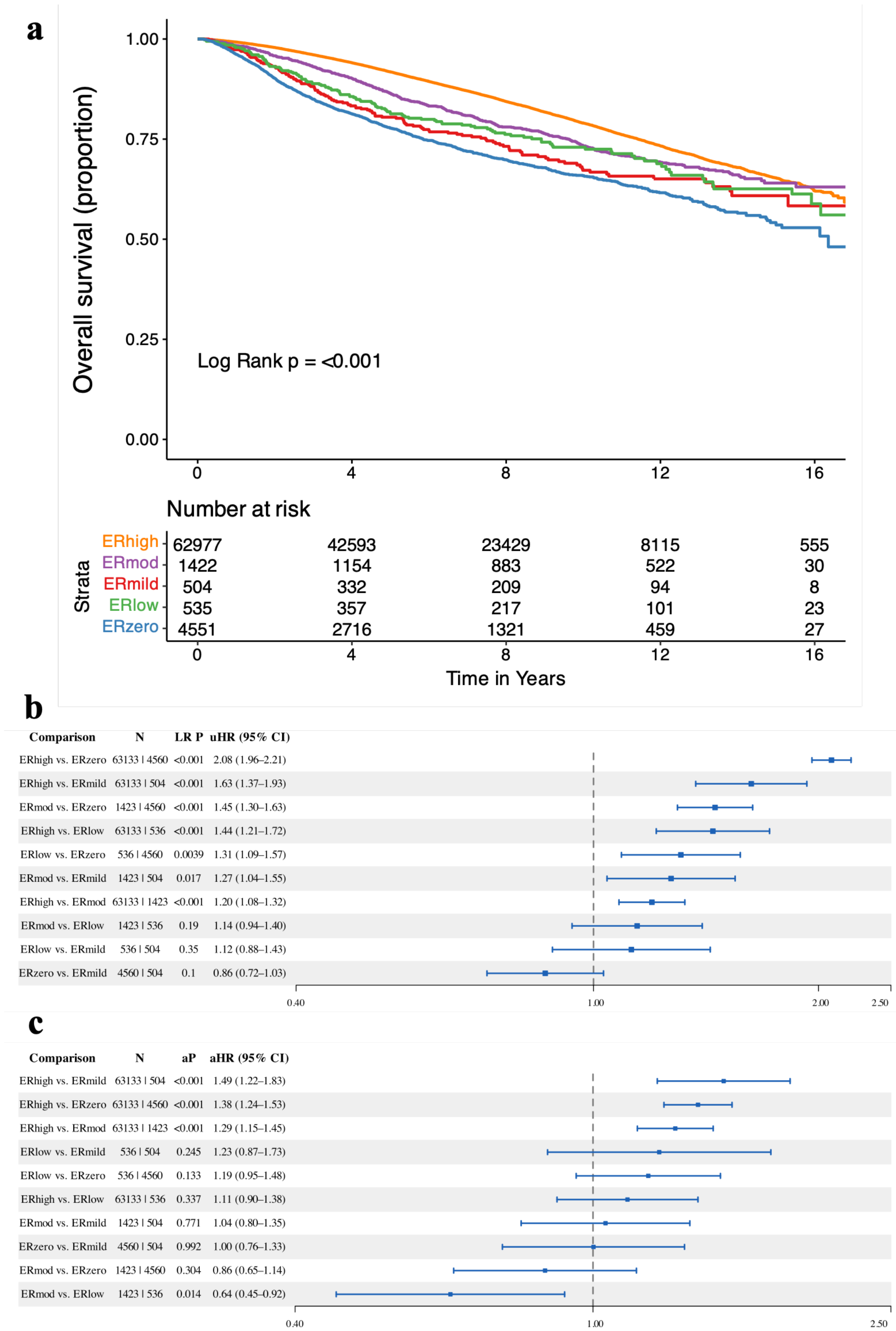

Figure S10 Overall survival analysis by ER subgroups in adjuvant setting (n = 70,156).

(a) Kaplan-Meier survival curves. (b) Forest plot of unadjusted hazard ratios (uHR). (c) Forest plot of adjusted hazard ratios (aHR) from a multivariable Cox proportional hazards model (Model 1), adjusted for tumor stage, grade, age at

diagnosis, year of diagnosis, and postoperative therapy. In all forest plots, the first group listed is the reference category. LR P, log-rank P value; aP, P value from the adjusted model; 95% CI, 95% confidence intervals.

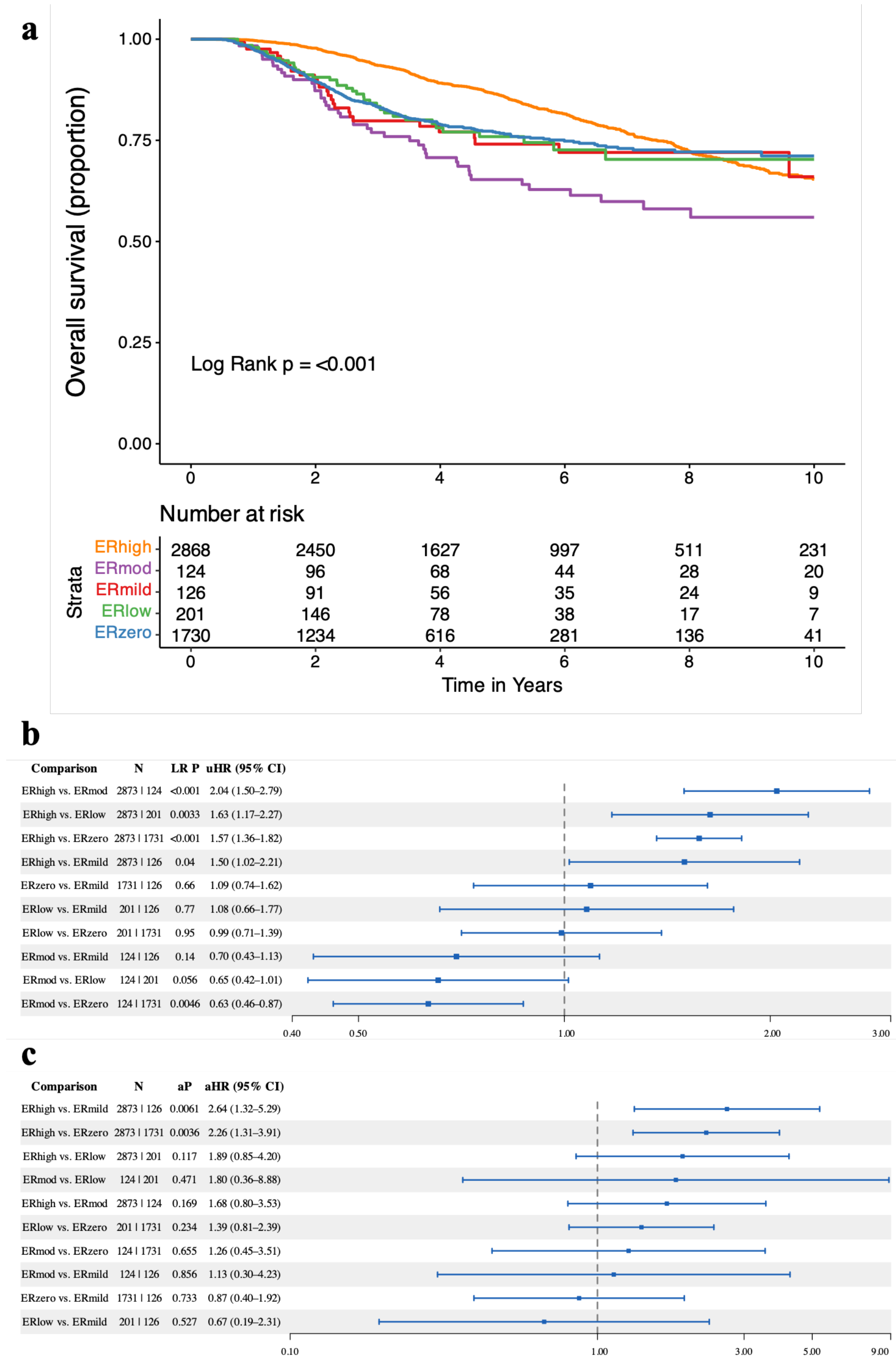

Figure S11 Overall survival analysis by ER subgroups in the neoadjuvant setting (n = 5055).

(a) Kaplan-Meier survival curves. (b) Forest plot of unadjusted hazard ratios (uHR). (c) Forest plot of adjusted hazard ratios (aHR) from a multivariable Cox model (Model 1), adjusted for tumor stage, grade, age at diagnosis, year of diagnosis, and postoperative therapy. The first group listed in each forest plot comparison serves as the reference. LR P, log-rank P value; aP, P value from the adjusted model; 95% CI, 95% confidence intervals.

Comparison	N	LR P	aP	aHR (95% CI)
ERhigh vs. ERmild	51069 ǀ 435	<0.001	<0.001	1.79 (1.40–2.29)
ERhigh vs. ERzero	51069 ǀ 5338	<0.001	<0.001	1.74 (1.55–1.96)
ERhigh vs. ERmod	51069 ǀ 603	<0.001	0.0018	1.40 (1.13–1.74)
ERlow vs. ERzero	554 ǀ 5338	0.024	0.0076	1.40 (1.09–1.79)
ERhigh vs. ERlow	51069 ǀ 554	<0.001	0.122	1.22 (0.95–1.57)
ERlow vs. ERmild	554 ǀ 435	0.46	0.461	1.18 (0.76–1.85)
ERmod vs. ERmild	603 ǀ 435	0.12	0.465	1.14 (0.81–1.60)
ERmod vs. ERzero	603 ǀ 5338	<0.001	0.726	1.07 (0.75–1.52)
ERzero vs. ERmild	5338 ǀ 435	0.29	0.537	0.90 (0.66–1.24)
ERmod vs. ERlow	603 ǀ 554	0.43	0.083	0.62 (0.36–1.06)

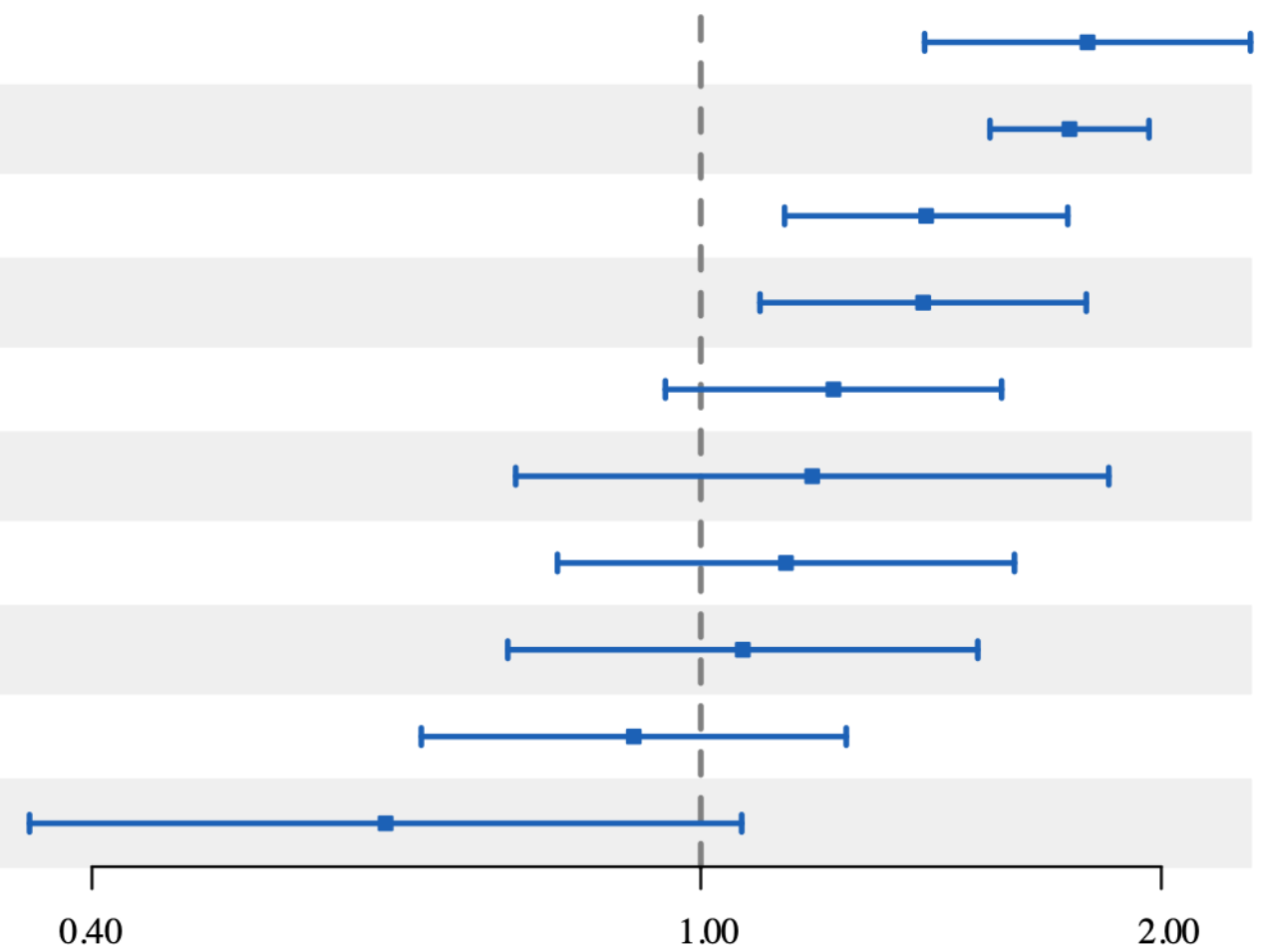

Figure S12 Multivariable analysis by ER subgroups, restricted to 2013–2023 (n = 57,871).

Forest plot showing adjusted hazard ratios (aHR) with 95% confidence intervals (95% CI) from the Cox proportional hazards model (Model 1). The first group listed in each comparison serves as the reference. Abbreviations: aHR, adjusted hazard ratio; 95% CI, 95% confidence interval.

a

Comparison	N	LR P	aP	aHR (95% CI)
ERhigh_CT+ET vs. ERmod_CT+ET	11247 ǀ 218	0.0016	<0.001	2.14 (1.47–3.11)
ERhigh_ETonly vs. ERmild_ETonly	30601 ǀ 147	<0.001	<0.001	2.08 (1.48–2.92)
ERhigh_CT+ET vs. ERmild_CT+ET	11247 ǀ 118	0.014	0.0068	2.04 (1.22–3.41)
ERmod_ETonly vs. ERmild_ETonly	147 ǀ 276	0.011	0.076	1.58 (0.95–2.62)
ERhigh_ETonly vs. ERmod_ETonly	30601 ǀ 276	0.0012	0.755	1.05 (0.77–1.42)
ERmod_CT+ET vs. ERmild_CT+ET	218 ǀ 118	0.97	0.839	0.93 (0.48–1.81)
ERzero_CTonly vs. ERlow_CTonly	2770 ǀ 250	0.51	0.168	0.78 (0.55–1.11)

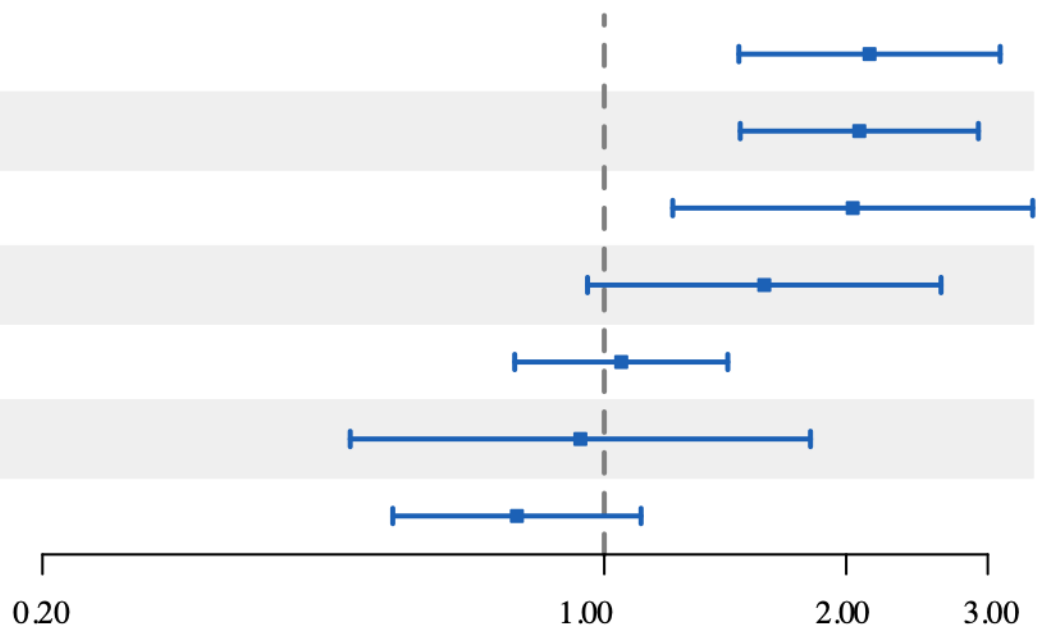

b

Comparison	N	LR P	aP	aHR (95% CI)
ERmod_ETonly vs. ERmod_CT+ET	218 ǀ 276	0.043	0.890	0.95 (0.49–1.87)
ERhigh_ETonly vs. ERhigh_CT+ET	11247 ǀ 30601	<0.001	<0.001	0.72 (0.66–0.79)
ERmild_ETonly vs. ERmild_CT+ET	147 ǀ 118	<0.001	0.106	0.52 (0.23–1.15)

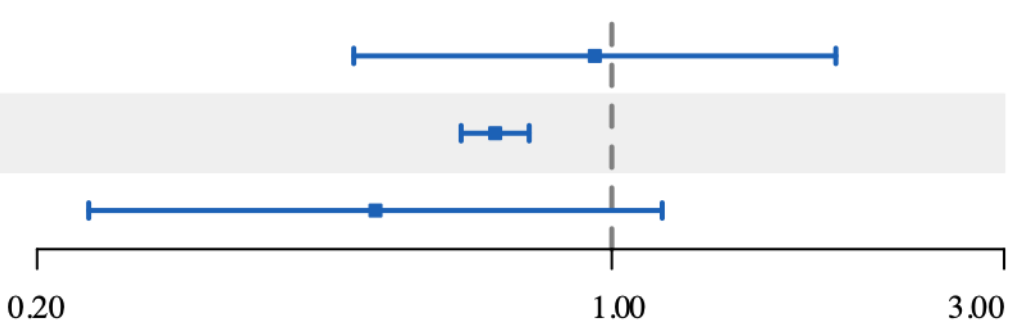

Figure S13 Multivariable analysis across ER subgroups by postoperative treatment, restricted to 2013–2023 (n = 57,871).

(a) Forest plot by different ER subgroups with the same treatment: The model was adjusted for tumor stage, grade, age, diagnosis year, and stratified by radiotherapy (Model 2). (b) Forest plot of different treatments within the ER subgroup, showing adjusted hazard ratio (aHR) for overall survival from Model 2. LR P, unadjusted log-rank p value; aP, adjusted p value; aHR, adjusted hazard ratios. Note: ERlow_CT+ET vs. ERlow_CTonly (in a), and ERlow_CT+ET vs. ERmod_CT+ET, ERlow_CT+ET vs. ERmild_CT+ET, ERhigh_CT+ET vs. ERlow_CT+ET (in b) were excluded because of extremely biased sample size.

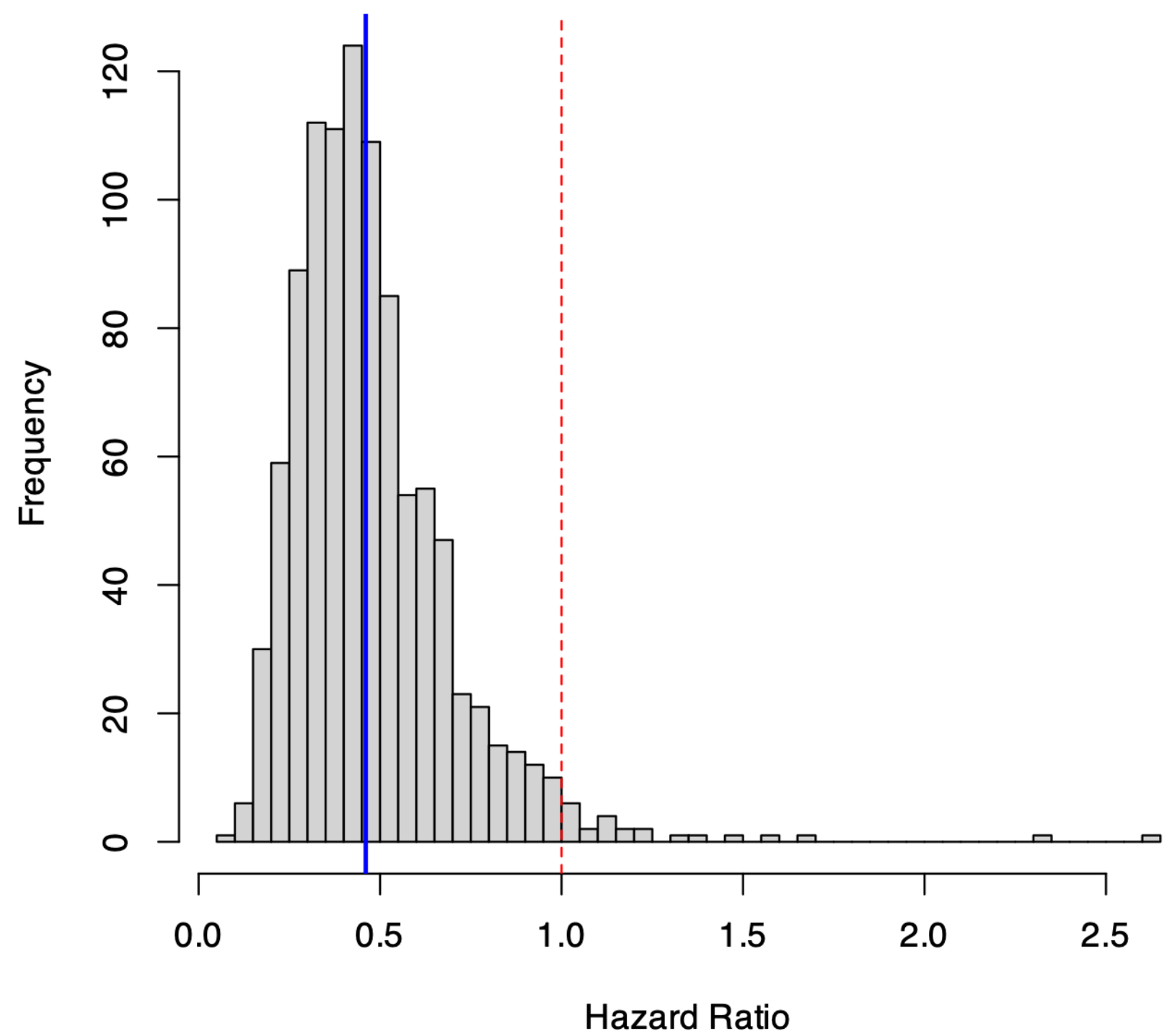

Figure S14 Bootstrap distribution of hazard ratios (ETonly vs. CT+ET) in ERmild tumors (n = 1000 resamples).

Histogram of 1,000 bootstrap hazard ratios (HR) estimates. The vertical blue line represents bootstrap-corrected HR (0.46), and the vertical dashed line indicates HR = 1. Bias-corrected 95% CI: 0.18–0.99.

ERgroup	no vs. pCR	LR P	uHR (95% CI)
ERzero	1161 | 570	<0.001	0.22 (0.15–0.32)
ERlow	128 | 73	<0.001	0.19 (0.07–0.53)
ERmild	95 | 31	0.011	0.12 (0.02–0.86)
ERmod	110 | 14	0.093	0.32 (0.08–1.31)
ERhigh	2783 | 90	0.1	0.54 (0.26–1.14)

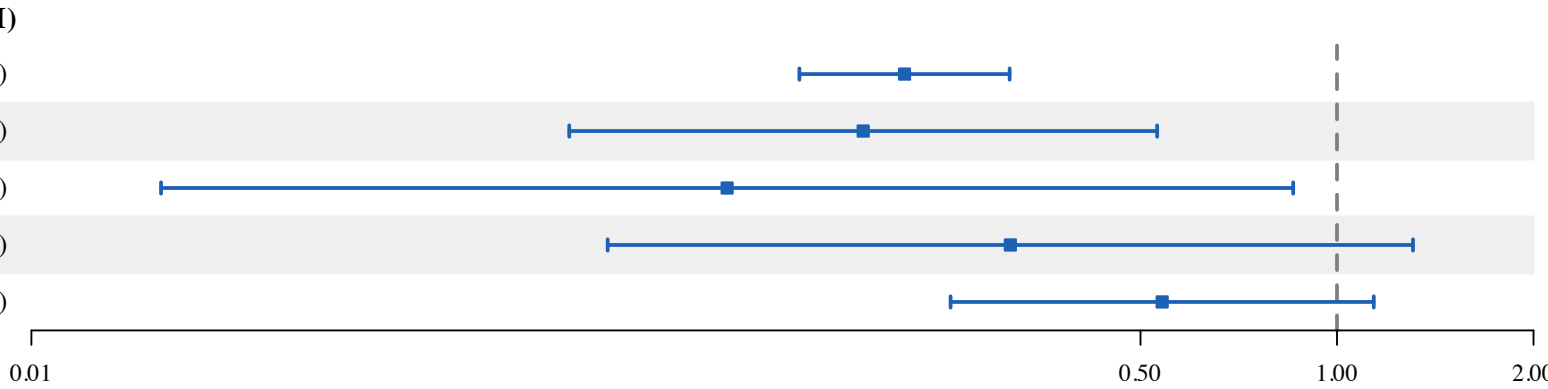

Figure S15 Impact of achieving a Pathologic Complete Response (pCR) on overall survival across ER subgroups following neoadjuvant therapy (n = 5055).

Unadjusted hazard ratios (uHR) and 95% confidence intervals (95% CI) compare the pCR group to the non-pCR reference group within each ER group. P values are from log-rank tests (LR P).

Table S1 Distribution of patient and tumor characteristics in the full cohort (n = 75,112).

Data are shown for the full population-based cohort of 75,112 women with breast cancer (diagnosed 2007–2023) and stratified by adjuvant (adj, n = 70,156) and neoadjuvant (neoadj, n = 5055) treatment settings.

Patient/tumor characteristics	adj<br>N = 70,156[1]	neoadj<br>N = 5,055[1]	full<br>N = 75,211[1]
**Age at Diagnosis**	65.0 (21.0, 102.0)	54.0 (23.0, 93.0)	64.0 (21.0, 102.0)
**Age at Diagnosis (group)**			
<40	1,693 (2.4%)	718 (14.2%)	2,411 (3.2%)
40-49	8,945 (12.8%)	1,234 (24.4%)	10,179 (13.5%)
50-64	23,390 (33.3%)	1,740 (34.4%)	25,130 (33.4%)
65-79	28,907 (41.2%)	1,138 (22.5%)	30,045 (39.9%)
≥80	7,221 (10.3%)	225 (4.5%)	7,446 (9.9%)
**Year of Diagnosis (Period)**			
2007-2012	16,912 (24.1%)	300 (5.9%)	17,212 (22.9%)
2013-2016	19,399 (27.7%)	1,152 (22.8%)	20,551 (27.3%)
2017-2020	19,082 (27.2%)	2,076 (41.1%)	21,158 (28.1%)
2021-2023	14,763 (21.0%)	1,527 (30.2%)	16,290 (21.7%)
**ER (median)**	100.0 (0.0, 100.0)	90.0 (0.0, 100.0)	100.0 (0.0, 100.0)
**ER subgroups**			
ERzero	4,560 (6.5%)	1,731 (34.2%)	6,291 (8.4%)
ERlow	536 (0.8%)	201 (4.0%)	737 (1.0%)
ERmild	504 (0.7%)	126 (2.5%)	630 (0.8%)
ERmod	1,423 (2.0%)	124 (2.5%)	1,547 (2.1%)
ERhigh	63,133 (90.0%)	2,873 (56.8%)	66,006 (87.8%)
**Progesterone receptor**			
Negative (<10%)	13,425 (19.1%)	2,579 (51.0%)	16,004 (21.3%)
Positive (≥10%)	55,617 (79.3%)	2,423 (47.9%)	58,040 (77.2%)
Unknown	1,114 (1.6%)	53 (1.0%)	1,167 (1.6%)

**Ki67 (median)**	18.0 (0.0, 100.0)	42.0 (0.0, 100.0)	20.0 (0.0, 100.0)
Unknown	13,157	301	13,458
**TNM stage (clin/path)**			
0	109 (0.2%)	3 (0.1%)	112 (0.1%)
I	41,487 (59.1%)	395 (7.8%)	41,882 (55.7%)
II	23,433 (33.4%)	3,714 (73.5%)	27,147 (36.1%)
III	4,833 (6.9%)	899 (17.8%)	5,732 (7.6%)
Unknown	294 (0.4%)	44 (0.9%)	338 (0.4%)
**Grade (NHG)**			
I	16,431 (23.4%)	290 (5.7%)	16,721 (22.2%)
II	38,489 (54.9%)	1,640 (32.4%)	40,129 (53.4%)
III	14,722 (21.0%)	842 (16.7%)	15,564 (20.7%)
Unknown	514 (0.7%)	2,283 (45.2%)	2,797 (3.7%)
**Lymph node metastases**			
1–3	13,853 (19.7%)	687 (13.6%)	14,540 (19.3%)
None	48,205 (68.7%)	1,614 (31.9%)	49,819 (66.2%)
Not available	92 (0.1%)	8 (0.2%)	100 (0.1%)
Unknown	8,006 (11.4%)	2,746 (54.3%)	10,752 (14.3%)
**Chemotherapy**			
Adjuvant	16,581 (23.6%)	0 (0.0%)	16,581 (22.0%)
Neoadj and postneoadj	0 (0.0%)	1,360 (26.9%)	1,360 (1.8%)
Neoadj only	0 (0.0%)	2,931 (58.0%)	2,931 (3.9%)
No	43,355 (61.8%)	479 (9.5%)	43,834 (58.3%)
Unknown	10,220 (14.6%)	285 (5.6%)	10,505 (14.0%)
**Endocrine therapy**			
Adjuvant	49,527 (70.6%)	0 (0.0%)	49,527 (65.9%)
Neoadj and postneoadj	0 (0.0%)	711 (14.1%)	711 (0.9%)
Neoadj only	0 (0.0%)	67 (1.3%)	67 (0.1%)
No	10,410 (14.8%)	4,225 (83.6%)	14,635 (19.5%)
Unknown	10,219 (14.6%)	52 (1.0%)	10,271 (13.7%)

**Combined adjuvant treatment**			
CT+ET	13,502 (19.2%)	725 (14.3%)	14,227 (18.9%)
ETonly	36,012 (51.3%)	2,209 (43.7%)	38,221 (50.8%)
CTonly	3,069 (4.4%)	677 (13.4%)	3,746 (5.0%)
None	7,335 (10.5%)	1,133 (22.4%)	8,468 (11.3%)
Unknown	10,238 (14.6%)	311 (6.2%)	10,549 (14.0%)
**Adjuvant radiotherapy**			
Yes	45,562 (64.9%)	4,216 (83.4%)	49,778 (66.2%)
No	14,383 (20.5%)	534 (10.6%)	14,917 (19.8%)
Unknown	10,211 (14.6%)	305 (6.0%)	10,516 (14.0%)
**Screening detected**			
Yes	37,985 (54.1%)	1,265 (25.0%)	39,250 (52.2%)
No	32,046 (45.7%)	3,780 (74.8%)	35,826 (47.6%)
Unknown	125 (0.2%)	10 (0.2%)	135 (0.2%)

[1]Median (Min, Max); n (%)

Abbreviations: ER subgroups, $ER^{0\%}$ (ERzero), $ER^{1–9\%}$ (ERlow), $ER^{10–29\%}$ (ERmild), $ER^{30–59\%}$ (ERmod), and $ER^{60–100\%}$ (ERhigh). Combined adjuvant treatment: CT+ET, chemotherapy and endocrine therapy; ETonly, endocrine therapy alone; CTonly, chemotherapy alone; None, neither chemotherapy nor endocrine therapy.

Note: "Not available" in Lymph node metastases indicates patients who received neoadjuvant treatment and achieved a pathologic complete response.

Table S2 Distribution of cohort and tumor characteristics in the SCAN-B subset (n = 4767).

Data are shown across five ER subgroups for the subset cohort of 4767 women with breast cancer from the SCAN-B cohort (diagnosed September 2010–May 2018).

**Cohort/tumor characteristics**	**ERzero**<br>N = 390[1]	**ERlow**<br>N = 57[1]	**ERmild**<br>N = 31[1]	**ERmod**<br>N = 38[1]	**ERhigh**<br>N = 4,251[1]
**Age at Diagnosis**	65.0 (30.0, 95.0)	70.0 (35.0, 95.0)	60.0 (35.0, 90.0)	65.0 (30.0, 95.0)	65.0 (25.0, 95.0)
**Age at Diagnosis (group)**					
<40	15 (3.8%)	2 (3.5%)	2 (6.5%)	1 (2.6%)	38 (0.9%)
40-49	42 (10.8%)	4 (7.0%)	5 (16.1%)	4 (10.5%)	297 (7.0%)
50-64	115 (29.5%)	13 (22.8%)	11 (35.5%)	12 (31.6%)	1,224 (28.8%)
65-79	130 (33.3%)	24 (42.1%)	8 (25.8%)	13 (34.2%)	1,963 (46.2%)
≥80	88 (22.6%)	14 (24.6%)	5 (16.1%)	8 (21.1%)	729 (17.1%)
**ER (median)**					
Negative	389 (99.7%)	57 (100.0%)	14 (45.2%)	0 (0.0%)	0 (0.0%)
Positive	1 (0.3%)	0 (0.0%)	17 (54.8%)	38 (100.0%)	4,251 (100.0%)
**Progesterone receptor**					
Negative	379 (97.2%)	54 (94.7%)	22 (71.0%)	24 (63.2%)	645 (15.2%)
Positive	11 (2.8%)	3 (5.3%)	9 (29.0%)	14 (36.8%)	3,606 (84.8%)
**TNM stage**					
I	174 (44.6%)	23 (40.4%)	10 (32.3%)	14 (36.8%)	2,114 (49.7%)
II	171 (43.8%)	28 (49.1%)	18 (58.1%)	16 (42.1%)	1,641 (38.6%)
III	36 (9.2%)	5 (8.8%)	1 (3.2%)	6 (15.8%)	361 (8.5%)
Unknown	9 (2.3%)	1 (1.8%)	2 (6.5%)	2 (5.3%)	135 (3.2%)
**Grade (NHG)**					
I	6 (1.5%)	0 (0.0%)	1 (3.2%)	4 (10.5%)	838 (19.7%)
II	55 (14.1%)	13 (22.8%)	9 (29.0%)	15 (39.5%)	2,474 (58.2%)
III	326 (83.6%)	44 (77.2%)	21 (67.7%)	19 (50.0%)	925 (21.8%)
Unknown	3 (0.8%)	0 (0.0%)	0 (0.0%)	0 (0.0%)	14 (0.3%)

[1]Median (Min, Max); n (%)

Abbreviations: ER subgroups, ER0% (ERzero), ER1–9% (ERlow), ER10–29% (ERmild), ER30–59% (ERmod), and ER60–100% (ERhigh). Note: "Not available" in Lymph node metastases indicates patients who received neoadjuvant treatment and achieved a pathologic complete response.

Table S3 Distribution of patient and tumor characteristics in the adjuvant setting by ER subgroups (n = 70,156).

Patient/tumor characteristics by ER subgroups in the adjuvant setting	ERzero	ERlow	ERmild	ERmod	ERhigh	p-value[2]
	N = 4,560[1]	N = 536[1]	N = 504[1]	N = 1,423[1]	N = 63,133[1]	
**Age at Diagnosis**	65.0 (21.0, 99.0)	65.0 (28.0, 95.0)	62.0 (24.0, 95.0)	61.0 (25.0, 99.0)	65.0 (21.0, 102.0)	<0.001
**Age at Diagnosis (group)**						<0.001
<40	275 (6.0%)	33 (6.2%)	38 (7.5%)	68 (4.8%)	1,279 (2.0%)	
40-49	576 (12.6%)	72 (13.4%)	98 (19.4%)	263 (18.5%)	7,936 (12.6%)	
50-64	1,334 (29.3%)	154 (28.7%)	137 (27.2%)	500 (35.1%)	21,265 (33.7%)	
65-79	1,591 (34.9%)	221 (41.2%)	167 (33.1%)	456 (32.0%)	26,472 (41.9%)	
≥80	784 (17.2%)	56 (10.4%)	64 (12.7%)	136 (9.6%)	6,181 (9.8%)	
**Year of Diagnosis (Period)**						<0.001
2007-2012	926 (20.3%)	174 (32.5%)	186 (36.9%)	920 (64.7%)	14,706 (23.3%)	
2013-2016	1,288 (28.2%)	155 (28.9%)	131 (26.0%)	253 (17.8%)	17,572 (27.8%)	
2017-2020	1,465 (32.1%)	121 (22.6%)	105 (20.8%)	140 (9.8%)	17,251 (27.3%)	
2021-2023	881 (19.3%)	86 (16.0%)	82 (16.3%)	110 (7.7%)	13,604 (21.5%)	
**Progesterone receptor**						<0.001
Negative (<10%)	4,418 (96.9%)	478 (89.2%)	278 (55.2%)	332 (23.3%)	7,919 (12.5%)	
Positive (≥10%)	136 (3.0%)	33 (6.2%)	185 (36.7%)	1,008 (70.8%)	54,255 (85.9%)	
Unknown	6 (0.1%)	25 (4.7%)	41 (8.1%)	83 (5.8%)	959 (1.5%)	
**Ki67 (median)**	60.0 (0.0, 100.0)	50.0 (0.0, 99.0)	37.0 (1.0, 95.0)	24.0 (0.0, 100.0)	17.0 (0.0, 100.0)	<0.001
Unknown	580	147	151	807	11,472	
**TNM stage (path)**						<0.001
0	4 (0.1%)	3 (0.6%)	0 (0.0%)	0 (0.0%)	102 (0.2%)	
I	2,231 (48.9%)	248 (46.3%)	223 (44.2%)	743 (52.2%)	38,042 (60.3%)	
II	1,911 (41.9%)	245 (45.7%)	233 (46.2%)	514 (36.1%)	20,530 (32.5%)	
III	395 (8.7%)	40 (7.5%)	43 (8.5%)	155 (10.9%)	4,200 (6.7%)	
Unknown	19 (0.4%)	0 (0.0%)	5 (1.0%)	11 (0.8%)	259 (0.4%)	
**Grade (NHG)**						<0.001
I	97 (2.1%)	44 (8.2%)	44 (8.7%)	318 (22.3%)	15,928 (25.2%)	
II	798 (17.5%)	140 (26.1%)	174 (34.5%)	669 (47.0%)	36,708 (58.1%)	
III	3,589 (78.7%)	342 (63.8%)	278 (55.2%)	427 (30.0%)	10,086 (16.0%)	
Unknown	76 (1.7%)	10 (1.9%)	8 (1.6%)	9 (0.6%)	411 (0.7%)	
**Lymph node metastases**						<0.001
1–3	640 (14.0%)	97 (18.1%)	86 (17.1%)	261 (18.3%)	12,769 (20.2%)	
None	3,037 (66.6%)	343 (64.0%)	317 (62.9%)	894 (62.8%)	43,614 (69.1%)	
Not available	9 (0.2%)	0 (0.0%)	0 (0.0%)	2 (0.1%)	81 (0.1%)	

Unknown	874 (19.2%)	96 (17.9%)	101 (20.0%)	266 (18.7%)	6,669 (10.6%)	
**Chemotherapy**						<0.001
Adjuvant	2,633 (57.7%)	277 (51.7%)	221 (43.8%)	413 (29.0%)	13,037 (20.7%)	
No	994 (21.8%)	134 (25.0%)	182 (36.1%)	725 (50.9%)	41,320 (65.4%)	
Unknown	933 (20.5%)	125 (23.3%)	101 (20.0%)	285 (20.0%)	8,776 (13.9%)	
**Endocrine therapy**						<0.001
Adjuvant	100 (2.2%)	39 (7.3%)	284 (56.3%)	1,017 (71.5%)	48,087 (76.2%)	
No	3,529 (77.4%)	372 (69.4%)	121 (24.0%)	121 (8.5%)	6,267 (9.9%)	
Unknown	931 (20.4%)	125 (23.3%)	99 (19.6%)	285 (20.0%)	8,779 (13.9%)	
**Combined adjuvant treatment**						<0.001
CT+ET	69 (1.5%)	13 (2.4%)	156 (31.0%)	396 (27.8%)	12,868 (20.4%)	
ETonly	31 (0.7%)	26 (4.9%)	126 (25.0%)	621 (43.6%)	35,208 (55.8%)	
CTonly	2,563 (56.2%)	263 (49.1%)	65 (12.9%)	17 (1.2%)	161 (0.3%)	
None	963 (21.1%)	108 (20.1%)	56 (11.1%)	104 (7.3%)	6,104 (9.7%)	
Unknown	934 (20.5%)	126 (23.5%)	101 (20.0%)	285 (20.0%)	8,792 (13.9%)	
**Adjuvant radiotherapy**						<0.001
Yes	2,641 (57.9%)	296 (55.2%)	285 (56.5%)	810 (56.9%)	41,530 (65.8%)	
No	989 (21.7%)	116 (21.6%)	120 (23.8%)	328 (23.0%)	12,830 (20.3%)	
Unknown	930 (20.4%)	124 (23.1%)	99 (19.6%)	285 (20.0%)	8,773 (13.9%)	
**Screening detected**						<0.001
Yes	1,583 (34.7%)	221 (41.2%)	209 (41.5%)	708 (49.8%)	35,264 (55.9%)	
No	2,966 (65.0%)	313 (58.4%)	295 (58.5%)	713 (50.1%)	27,759 (44.0%)	
Unknown	11 (0.2%)	2 (0.4%)	0 (0.0%)	2 (0.1%)	110 (0.2%)	

[1]Median (Min, Max); n (%)

[2]Kruskal-Wallis rank sum test; Pearson's Chi-squared test with simulated p-value (based on 2000 replicates); Fisher's Exact Test for Count Data with simulated p-value (based on 2000 replicates)

Abbreviations: ER subgroups, $ER^{0\%}$ (ERzero), $ER^{1–9\%}$ (ERlow), $ER^{10–29\%}$ (ERmild), $ER^{30–59\%}$ (ERmod), and $ER^{60–100\%}$ (ERhigh). Combined adjuvant treatment: CT+ET, chemotherapy and endocrine therapy; ETonly, endocrine therapy alone; CTonly, chemotherapy alone; None, neither chemotherapy nor endocrine therapy.

Note: "Not available" in Lymph node metastases indicates patients who received neoadjuvant treatment and achieved a pathologic complete response.

Table S4 Distribution of patient and tumor characteristics in the neoadjuvant setting by ER subgroups (n = 5055).

Patient/tumor characteristics by ER subgroups in the neoadjuvant setting	ERzero	ERlow	ERmild	ERmod	ERhigh	p-value[2]
	N = 1,731[1]	N = 201[1]	N = 126[1]	N = 124[1]	N = 2,873[1]	
**Age at Diagnosis**	53.0 (23.0, 86.0)	53.0 (25.0, 87.0)	49.0 (23.0, 81.0)	52.0 (23.0, 92.0)	56.0 (23.0, 93.0)	<0.001
**Age at Diagnosis (group)**						<0.001
<40	319 (18.4%)	43 (21.4%)	29 (23.0%)	25 (20.2%)	302 (10.5%)	
40-49	430 (24.8%)	45 (22.4%)	38 (30.2%)	27 (21.8%)	694 (24.2%)	
50-64	577 (33.3%)	70 (34.8%)	38 (30.2%)	46 (37.1%)	1,009 (35.1%)	
65-79	387 (22.4%)	38 (18.9%)	20 (15.9%)	20 (16.1%)	673 (23.4%)	
≥80	18 (1.0%)	5 (2.5%)	1 (0.8%)	6 (4.8%)	195 (6.8%)	
**Year of Diagnosis (Period)**						<0.001
2007-2012	27 (1.6%)	9 (4.5%)	9 (7.1%)	24 (19.4%)	231 (8.0%)	
2013-2016	279 (16.1%)	39 (19.4%)	36 (28.6%)	42 (33.9%)	756 (26.3%)	
2017-2020	771 (44.5%)	79 (39.3%)	41 (32.5%)	37 (29.8%)	1,148 (40.0%)	
2021-2023	654 (37.8%)	74 (36.8%)	40 (31.7%)	21 (16.9%)	738 (25.7%)	
**Progesterone receptor**						<0.001
Negative (<10%)	1,690 (97.6%)	190 (94.5%)	101 (80.2%)	72 (58.1%)	526 (18.3%)	
Positive (≥10%)	39 (2.3%)	11 (5.5%)	20 (15.9%)	46 (37.1%)	2,307 (80.3%)	
Unknown	2 (0.1%)	0 (0.0%)	5 (4.0%)	6 (4.8%)	40 (1.4%)	
**Ki67 (median)**	70.0 (0.0, 100.0)	70.0 (5.0, 100.0)	75.0 (5.0, 100.0)	50.0 (5.0, 100.0)	30.0 (1.0, 100.0)	<0.001
Unknown	43	6	7	23	222	
**TNM stage (clin)**						<0.001
0	0 (0.0%)	0 (0.0%)	0 (0.0%)	0 (0.0%)	3 (0.1%)	
I	173 (10.0%)	10 (5.0%)	10 (7.9%)	6 (4.8%)	196 (6.8%)	
II	1,336 (77.2%)	162 (80.6%)	90 (71.4%)	88 (71.0%)	2,038 (70.9%)	
III	207 (12.0%)	27 (13.4%)	26 (20.6%)	29 (23.4%)	610 (21.2%)	
Unknown	15 (0.9%)	2 (1.0%)	0 (0.0%)	1 (0.8%)	26 (0.9%)	
**Grade (NHG)**						<0.001
I	16 (0.9%)	1 (0.5%)	1 (0.8%)	3 (2.4%)	269 (9.4%)	
II	328 (18.9%)	36 (17.9%)	13 (10.3%)	25 (20.2%)	1,238 (43.1%)	
III	486 (28.1%)	53 (26.4%)	37 (29.4%)	21 (16.9%)	245 (8.5%)	

Unknown	901 (52.1%)	111 (55.2%)	75 (59.5%)	75 (60.5%)	1,121 (39.0%)	
**Lymph node metastases**						<0.001
1–3	140 (8.1%)	23 (11.4%)	10 (7.9%)	15 (12.1%)	499 (17.4%)	
None	842 (48.6%)	86 (42.8%)	49 (38.9%)	22 (17.7%)	615 (21.4%)	
Not available	2 (0.1%)	0 (0.0%)	0 (0.0%)	1 (0.8%)	5 (0.2%)	
Unknown	747 (43.2%)	92 (45.8%)	67 (53.2%)	86 (69.4%)	1,754 (61.1%)	
**Chemotherapy**						<0.001
Neoadj and postneoadj	603 (34.8%)	63 (31.3%)	34 (27.0%)	29 (23.4%)	631 (22.0%)	
Neoadj only	950 (54.9%)	120 (59.7%)	85 (67.5%)	79 (63.7%)	1,697 (59.1%)	
No	0 (0.0%)	2 (1.0%)	1 (0.8%)	8 (6.5%)	468 (16.3%)	
Unknown	178 (10.3%)	16 (8.0%)	6 (4.8%)	8 (6.5%)	77 (2.7%)	
**Endocrine therapy**						<0.001
Neoadj and postneoadj	10 (0.6%)	2 (1.0%)	6 (4.8%)	11 (8.9%)	682 (23.7%)	
Neoadj only	28 (1.6%)	1 (0.5%)	1 (0.8%)	1 (0.8%)	36 (1.3%)	
No	1,686 (97.4%)	197 (98.0%)	118 (93.7%)	109 (87.9%)	2,115 (73.6%)	
Unknown	7 (0.4%)	1 (0.5%)	1 (0.8%)	3 (2.4%)	40 (1.4%)	
**Combined adjuvant treatment**						<0.001
CT+ET	25 (1.4%)	5 (2.5%)	16 (12.7%)	25 (20.2%)	654 (22.8%)	
ETonly	33 (1.9%)	14 (7.0%)	54 (42.9%)	82 (66.1%)	2,026 (70.5%)	
CTonly	578 (33.4%)	58 (28.9%)	18 (14.3%)	4 (3.2%)	19 (0.7%)	
None	917 (53.0%)	107 (53.2%)	32 (25.4%)	5 (4.0%)	72 (2.5%)	
Unknown	178 (10.3%)	17 (8.5%)	6 (4.8%)	8 (6.5%)	102 (3.6%)	
**Adjuvant radiotherapy**						<0.001
Yes	1,398 (80.8%)	160 (79.6%)	107 (84.9%)	106 (85.5%)	2,445 (85.1%)	
No	157 (9.1%)	24 (11.9%)	13 (10.3%)	12 (9.7%)	328 (11.4%)	
Unknown	176 (10.2%)	17 (8.5%)	6 (4.8%)	6 (4.8%)	100 (3.5%)	
**Screening detected**						<0.001
Yes	360 (20.8%)	43 (21.4%)	20 (15.9%)	18 (14.5%)	824 (28.7%)	
No	1,367 (79.0%)	158 (78.6%)	106 (84.1%)	106 (85.5%)	2,043 (71.1%)	
Unknown	4 (0.2%)	0 (0.0%)	0 (0.0%)	0 (0.0%)	6 (0.2%)	
**Pathologica complete response (pCR)**						<0.001
no pCR	1,161 (67.1%)	128 (63.7%)	95 (75.4%)	110 (88.7%)	2,783 (96.9%)	
pCR	570 (32.9%)	73 (36.3%)	31 (24.6%)	14 (11.3%)	90 (3.1%)	

[1]Median (Min, Max); n (%)

[2]Kruskal-Wallis rank sum test; Pearson's Chi-squared test with simulated p-value

(based on 2000 replicates); Fisher's Exact Test for Count Data with simulated p-value

(based on 2000 replicates)
Abbreviations: ER subgroups, $ER^{0\%}$ (ERzero), $ER^{1–9\%}$ (ERlow), $ER^{10–29\%}$ (ERmild), $ER^{30–59\%}$ (ERmod), and $ER^{60–100\%}$ (ERhigh). Combined adjuvant treatment: CT+ET, chemotherapy and endocrine therapy; ETonly, endocrine therapy alone; CTonly, chemotherapy alone; None, neither chemotherapy nor endocrine therapy.
Note: "Not available" in Lymph node metastases indicates patients who received neoadjuvant treatment and achieved a pathologic complete response.

Table S5 The distributions of the incidence of five ER subgroups by diagnostic period.

Cohort/tumor characteristics	2007-2012<br>N = 17,212[1]	2013-2016<br>N = 20,551[1]	2017-2020<br>N = 21,158[1]	2021-2023<br>N = 16,290[1]
**ER subgroups**				
ERzero	953 (5.5%)	1,567 (7.6%)	2,236 (10.6%)	1,535 (9.4%)
ERlow	183 (1.1%)	194 (0.9%)	200 (0.9%)	160 (1.0%)
ERmild	195 (1.1%)	167 (0.8%)	146 (0.7%)	122 (0.7%)
ERmod	944 (5.5%)	295 (1.4%)	177 (0.8%)	131 (0.8%)
ERhigh	14,937 (86.8%)	18,328 (89.2%)	18,399 (87.0%)	14,342 (88.0%)

[1]n (%)

Table S6 The assessment of events per variable (EPV) across five ER subgroups.

ER subgroups	n	Events	Event_rate	EPV_per_stratum[1]
ERhigh	66,006	9,985	0.15	624.06
ERmod	1,547	436	0.28	27.25
ERmild	630	161	0.26	10.06
ERlow	737	168	0.23	10.50
ERzero	6,291	1,518	0.24	94.88

[1]events-per-variable (EPV) assessment

Table S7 The assessment of events per variable (EPV) across five ER subgroups by postoperative treatment.

ER subgroups	n	Events	Event_rate	EPV_per_stratum[1]
ERhigh_CT+ET	13,522	1,354	0.10	96.71
ERhigh_ETonly	37,234	5,882	0.16	420.14
ERlow_CTonly	321	61	0.19	4.36
ERmild_CT+ET	172	25	0.15	1.79
ERmild_ETonly	180	57	0.32	4.07
ERmod_CT+ET	421	87	0.21	6.21
ERmod_ETonly	703	218	0.31	15.57
ERzero_CTonly	3,141	547	0.17	39.07

[1]events per variable (EPV) assessment
